# CRISPR/Cas9-mediated targeted knock-in of large constructs using nocodazole and RNase HII

**DOI:** 10.1038/s41598-023-29789-1

**Published:** 2023-02-15

**Authors:** Shahin Eghbalsaied, Wilfried A. Kues

**Affiliations:** 1 Biotechnology/Stem Cell Physiology, Friedrich-Loeffler-Institut (FLI), Federal Research Institute for Animal Health, Höltystr. 10, 31535 Neustadt, Germany; 2 Department of Animal Science, Isfahan (Khorasgan) Branch, Islamic Azad University, Tehran, Iran; 3 School of Biosciences, Royal Parade, The University of Melbourne, Melbourne, VIC Australia

**Keywords:** Molecular engineering, DNA recombination, DNA

## Abstract

On-target integration of large cassettes via homology-directed repair (HDR) has several applications. However, the HDR-mediated targeted knock-in suffered from low efficiency. In this study, we made several large plasmids (12.1–13.4 kb) which included the CRISPR/Cas9 system along with a puromycin transgene as part of the large DNA donor (5.3–7.1 kb insertion cassettes) and used them to evaluate their targeted integration efficiency into a transgenic murine embryonic fibroblast (MEF) cell line carrying a single copy of a Venus transgene. We established a detection assay by which HDR events could be discriminated from the error-prone non-homologous end-joining (NHEJ) events. Improving the plasmid quality could considerably leverage the cell toxicity impediment of large plasmids. The use of the TILD (targeted integration with linearized dsDNA) cassettes did not improve the HDR rate compared to the circular plasmids. However, the direct inclusion of nocodazole into the electroporation solution significantly improved the HDR rate. Also, simultaneous delivery of RNase HII and the donor plasmids into the electroporated cells considerably improved the HDR events. In conclusion, the results of this study showed that using cell synchronization reagents in the electroporation medium can efficiently induce HDR rate in the mammalian genome.

## Introduction

Implementation of the CRISPR (clustered regularly interspaced short palindromic repeats)/Cas9 (CRISPR-associated protein 9) nuclease system has been widely used for targeted deletion and insertion of short fragments in the genomic DNA^1^. Using ‘all-in-one’ CRISPR vectors, expressing both guide RNAs and Cas9 nuclease, is a straight-forward and highly efficient tool for CRISPR-mediated genome editing in mammalian species^1,2^. Although with low efficiency, targeted knock-in of short amplicons, mainly for precise substitution of single nucleotides, has been achieved via CRISPR/Cas9^3^. However, basic transgenesis which is defined as transferring an exogenous gene to an organism^4^, is still challenging by CRISPR approaches^5^. Targeted insertion of large cassettes via homology-directed repair (HDR) have several applications. For basic sciences, it is useful to assess the functionality of large chromosomic regions and DNA contigs, as well as making a CRISPR/Cas9 encoding allele for gene drive systems. It has been considered that HDR-mediated targeted knock-in is inefficient and associated with high rate of insertions/deletions (indels). Therefore, dCas9- and nCas9 fused proteins were developed for the efficient base-editing and prime-editing technologies^6,7^. However, both technologies are restricted to single nucleotide or short sequence edition. Achieving the aim of on-target large insertion cassettes, a technology has to be devised to efficiently transfer large constructs into cells with a low rate of cell toxicity, and efficiently induce the HDR event^8^.

Double strand breaks (DSBs), the prerequisite for homologous recombination, occur during S and M phases of cell cycle^9^. Therefore cell synchronization has been considered as an effective approach to improve the HDR efficiency. Nocodazole pretreatment of cells increased the rate of G2-M phase up to 80%^10^. Pretreatment of different cell types with nocodazole substantially increased the HDR rate using single strand and double strand DNA donors involving short insertion cassettes^10,11^.

RNase HII activity has been detected as an important requirement for both R-loop processing and ribonucleotide excision repair in eukaryotes^12–14^. Interestingly, the highest expression of RNase HII was observed in S phase and G2-M phase^15^. High sensitivity of RNase H depleted yeasts to DSBs, a common phenomenon with Rad51 and Rad52 knock-out strains, suggested the importance of RNase H in HR-mediated DSB repair^16^. It has been speculated that the presence of high amounts of RNA–DNA hybrids during DNA repair in RNase HII-deficient yeasts can inhibit the completion of the HR repair process^16^. In agreement with this hypothesis, RNase HII-mediated degradation of LINE-1 (Long Interspersed Nuclear Element-1) RNAs and lncRNAs (long noncoding RNAs) from the DNA–RNA hybrids is a required step for efficient LINE-1 retrotransposition and the control of genome stability and epigenome shaping, respectively^17,18^. However, the importance of RNase HII on CRISPR/Cas9-mediated HDR events is yet to be evaluated.

In this study, we targeted a Venus allele in a transgenic mouse cell line. We developed an electroporation technology for efficient and gentle delivery of large plasmids (ranging from 12.0 to 13.4 kb) with insertion cassettes (ranging from 5.3 to 7.1 kb) We used a hemizygous cell line that carried only a single allele of the Venus transgene. The hemizygous transgenic cell line guaranteed a direct detection assay for on-target KI events. We supplemented nocodazole and RNase HII in the electroporation media, and found a high association between the nocodazole/RNase HII dose and the HDR efficiency.

## Materials and methods

### Materials

All plastic consumables including cell culture flasks and plates, tubes, and filter tips were purchased from Sarstedt AG & Co. (Germany). Chemical reagents were purchased as follows: Dulbecco’s Phosphate Buffered Saline without calcium chloride and magnesium chloride (#D6662-10X1L, Sigma-Aldrich, Germany); Opti-MEM 1X + GlutaMAX, Reduced serum medium (#1854076, Gibco, Life Technologies, Germany); DMEM High Glucose (4.5 g/l) w/o l-glutamine (#DMEM-HXA, Capricorn Scientific GmbH, Germany); l-glutamine for cell culture (#A3704,0100, Applichem, Germany); MEM nonessential amino acids solution (100x) (#NEAA-B, Capricorn Scientific GmbH, Germany); 2-mercaptoethanol (#BCBS5481, Sigma-Aldrich, Germany); penicillin/streptomycin solution (100x) (#PS-B, Capricorn Scientific GmbH, Germany); Trypsin–EDTA (10×) (#L11-003, GE Healthcare, PPA Laboratories GmbH, Austria); fetal bovine serum (#10270-106, Gibco, ThermoFisherScientific, Germany); dimethyl sulfoxide (#D4540-500ML, Sigma-Aldrich, Germany); LIF (hBA-FL) (#sc-4377, Santa Cruz Biotechnology, Germany); sodium pyruvate (#P2256, Sigma-Aldrich, Germany); gelatin from bovine skin (#G939-100G, Sigma-Aldrich, Germany); and Hoechst 33354 (#62249, Thermo Scientific, Germany). We used a Gene Pulser Xcell system with CE Module from BIO-RAD (Germany) and 4 mm electroporation cuvettes (#748052, Biozym, Germany).

### Plasmids

#### Modified pX459 plasmids expressing Cas9 and gRNA

We modified the pX459 plasmid (9151 bp), which encodes a Cas9 protein followed by puromycin resitance gene under CAGGS promoter/enhancer, and expresses the gRNA scaffold under the U6 promoter. Briefly, three gRNAs were selected to target the CAGGS promoter (− 252, − 72, and − 69) and one gRNA which targets the 5´coding part (+ 121) of the Venus cDNA (Table S1).We used the Farnham method for cloning of the double stranded oligonucleotides into the pX459 plasmid^19^. The efficacy of the gRNAs, in the pX459 plasmid, was previously shown for making indels in the target sites^1^. In summary, gRNAs targeting the promoter region had no impact on the Venus expression, while the ORF-targeting gRNA efficiently knocked-out the Venus expression.

#### Large plasmids carrying the CRISPR/Cas9 and gRNA cassette

The characteristics details of all large plasmids, which were named pSGDs, are provided in Table S2. Plasmid size ranged from 12.0 to 13.4 kb and the insertion cassette ranged from 5.3 to 7.1 kb. The pSGD plasmids had a left homology arm (LHA) of 1.7–2.1 kb and right homology arm (RHA) of 1.2–1.3 kb with the Venus transgene. For all pSGDs, the insertion cassette that was located between LHA and RHA included the sequence of one or two gRNAs each under U6 promoter as well as the T2A-linked sequences of wild type Cas9 protein and puromycin resistance. The cloning details of the pSGD plasmids are provided in the Supplementary Methods and the accessory sequences for the gene cloning are provided in Table S3. For all plasmids, after the restriction digestion, the reaction was inactivated at 80 °C for 10 min. All cloning steps were carried out using 1 µl of the T4 DNA ligase in 20 µl reaction volume. Ligations were carried out for 2 h at room temperature. We cloned the pSGD62 and pSGD74 plasmids with a similar size (13.4 kb) carrying the insertion cassette including human lysozyme cDNA, gRNA, Cas9, and puromycin as well as left and right homology arms. Then, the lysozyme fragment was removed from both pSGD62 and pSGD74 plasmids to make pSGD52 and pSGD73 plasmids, having a similar size of 12.4 kb. All the above-mentioned plasmids included either of gRNA-252, -72, and -69 that their NHEJ-produced indels had no effect on the Venus expression. However, as a positive control for the transfection, we constructed the pSGD75 carrying gRNA + 121 that targeted the beginning part of the Venus coding sequence. Subsequently, the transfected cells with the gRNA + 121 were completely Venus knock-out either via NHEJ-produced indels or HDR-mediated knock-in events. To make the pSGD75 plasmid, the lysozyme cDNA and gRNA-72 were removed to combine the CMV promoter with the hybrid intron of Cas9 promoter. Instead, a synthetic construct carrying both gRNA-72 and gRNA + 121 was inserted following the Cas9-puromycin DNA. The new construct was the largest plasmid. Then, the gRNA + 121 which was between two *Sfu* I restriction sites was removed to make the pSGD77 plasmid. The gRNA-72 segment was replaced with that of gRNA-69 to make pSGD69 plasmid. Therefore, neither of pSGD69 and pSGD77 carried the gRNA + 121 that its NHEJ-produced indels could similarly knock-out the Venus expression as the HDR-mediated knock-in did.

### Ethics approval, cell cultures and electroporation condition

The German legislation (Tierschutzgesetz §4, Abs. 3) does not require an ethics approval for humanely killing of laboratory animals for the purpose of organ removal. In all cases, the local animal welfare officer approves and enumerates all euthanasia of animals and verifies that all methods have been performed in accordance with relevant policies and regulations.

We used mouse embryonic fibroblast cells (MEFs), carrying either no- or a single-copy of the Venus transgene (Venus +/−), which were bred in the mouse house of the institute (1). Mouse embryonic fibroblast cells were isolated from day 11 embryos of the Venus −/− (wild type) or hemizygous Venus +/− embryos. Electroporation was carried out with a Bio-Rad electroporation device. We used the electroporation protocol which we’ve recently developed for electroporation of CRISPR/Cas9 plasmids^1^. In summary, the protocol used 20 µg of plasmid DNA in 4 mm cuvettes, with the following electroporation program: square-wave protocol with 300 V, each 10 ms pulse length, 2 pulses, and 10 s pulse interval. After the electroporation process, cells were transferred into culture medium in 6-well plates and incubated at 37 °C and 5% CO_2_.

### Determination of the HDR versus NHEJ events

We used MEFs cells with a single-copy of Venus for making KO-Venus cells. To establish a discriminative protocol for HDR-mediated knock-in versus NHEJ-produced indels, we used pSGD52 that involved gRNA-252 as well as the Cas9-puromycin expressing cassette. We selected gRNA-252 targeting the promoter region of the Venus so that a targeted disruption by small indels had no effect on the Venus expression (for more details about the gRNAs, please see^1^). Therefore, an on-target knock-in was only the case if the Venus expression was silenced. The transfection protocol was as follows: first, we electroporated the gRNA expressing plasmid into the MEF cells carrying a single-copy of Venus based on the mentioned protocol in the previous section. The medium was changed 6–8 h after the electroporation with a puromycin-supplemented medium (1000 ng/ml). To exclude non-transfected cells from the analysis, the puromycin selection was carried out for two successive days so that all cells in the non-transfected group died completely. The remaining cell population contained the pSGD plasmids and therefore underwent either of NHEJ or HDR events. The culture of these cells continued to day 10. To discriminate the HDR-mediated on-target events, we devised a system with two detectable markers for HDR events. First, the Venus knock-out was only visible when the promoter was disrupted by large insertions. Small indels in the promoter region had no effect on the Venus expression. This piece of evidence was assessed 10 days following the electroporation to avoid possible interference of remnant fluorescent protein. To confirm the gRNA activity, a fraction of electroporated cells were lysed and their genomic DNA was used for PCR (primer list is included in Table S4). The PCR products were sequenced and analyzed using the TIDE (Tracking of Indels by Decomposition) webtool (http://shinyapps.datacurators.nl/tide/). The rate of Venus knock-out cells from the transfected cells was considered as the HDR-mediated targeted knock-in. Off-target sequences of gRNAs on the mouse genome was predicted using the online webtool of Cas-OFFinder (http://www.rgenome.net/cas-offinder/), considering up to 3 mismatches (Table S5)^20^.

Since the insertion cassette included a puromycin resistance gene, the Venus knock-out population must also be resistant to puromycin. A fraction of day 10 cells was further used for puromycin selection process. We observed puromycin resistance in the electroporated cells though some of them maintained the Venus fluorescence. We assumed this attribute was because of the episomal presence of large constructs without the HDR event. This could produce a confounding effect between the fraction of cells carrying the HDR-mediated on-target knock-in cassettes and the rest of electroporated population that had the vector in episomal state and subsequently associated with NHEJ indels events. To avoid this, we prolonged the culture of electroporated cells for 2–3 months, changing the puromycin-supplemented culture medium each two days, so that the remained cells were both puromycin resistant and Venus knock-out. The HDR-mediated targeted disruption of the Venus promoter by the SGD construct caused a 5.3-kb insertion into the Venus CAGGS promoter. Amplification of these large fragments was not mediated by routine Taq DNA polymerases. Overall, the results of PCR, fluorescence microscopy, and flow cytometry (details of the procedures are provided in next sections) showed that the Venus knock-out events using pSGD52 construct carrying one of the gRNA-252 was due to the HDR event. Therefore, we considered Venus knock-out rate using the SGD cassettes as the HDR rate.

### Venus knock-outs using HDR by SGD constructs

In addition to pSGD52, a series of six SGD constructs all with left and right homology arms for the CAGGS promoter and the Venus transgene were electroporated into the Venus +/− cells. All pSGDs carried the insertion cassette of 5.9 to 7.1 kb, including Cas9 and puromycin resistance gene under the CMV promoter. Two pSGD plasmids (pSGD62 and pSGD74) involved the lysozyme transgene along with the Cas9-Puromycin genes. All plasmids carried either of gRNA-69 and gRNA-72 that targeted the Venus promoter and had no effect on the Venus expression in NHEJ-produced indels conditions. However, as a positive control for the transfection, we constructed the pSGD75 carrying gRNA + 121 that targeted the beginning part of the Venus coding sequence. Subsequently, the transfected cells with the gRNA + 121 were completely Venus knock-out either via NHEJ-produced indels or HDR-mediated knock-in events. A 2-day puromycin selection was carried out 6–8 h following the transfection. Then, the media was changed two-daily without puromycin selection. Cell culture continued to day 10 and a fraction of cells were screened for the Venus expression by fluorescent microscopy and FACS analysis. A fraction of cells from each group were cultured in the media supplemented with puromycin for a period of 2–3 months, exchanging the medium each 2 days, to select only cells with the HDR-mediated integrated cassettes. DNA was extracted from the electroporated cells using the salting-out method, and the SGD-HDR events were quantified using the digital PCR technique (details are provided in next sections). A HEX-labelled probe was designed and synthetized by IDT (Integrated DNA Technologies, USA) which cover the Cas9 transgene, and a FAM-labelled probe which target the Venus part was used as the housekeeping gene. The rate of Cas9/Venus copy number was calculated afterwards. To confirm the integration of the SGD construct via HDR, a long amplification PCR was carried out for the whole and left and right ends of fragment. The details of PCRs are provided in next sections.

### Targeted integration of linearized double-stranded DNA (TILD)

All SGD constructs had the *BamH1* restriction sequence at the 5′ and 3′ LTRs. The constructs were digested with *BamHI* enzyme (ThermoFisher Scientific) to make the TILD fragments^3^. Electroporation of TILD fragments were carried out with the corresponding gRNA encoding pX459 plasmid. In addition, considering the nocodazole effect on the HDR efficiency of TILD fragments, cells were cultured in the nocodazole-supplemented medium (700 ng/ml) for 15 h and were divided into two groups of the gRNA-encoding circular pSGD plasmid and the TILD fragments combined with the gRNA-encoding pX459 plasmid. It has been shown that linearized plasmids have considerably lower expression of transgene compared to the circular plasmid^21,22^. To provide a similar condition for appropriate expression of required gRNAs, circular gRNA-expressing pX459 plasmid was co-electroporated with the TILD fragment. For each TILD and pX459 construct, 20 µg were co-electroporated into the MEF cells. Eight hours following the electroporation, the electrotransfected cells were selected for puromycin (1000 ng/ml) for 24 h.

### Supplementation of nocodazole and RNase HII in the electroporation media

Cells were cultivated in the D10 medium supplemented with nocodazole (#M1404, Sigma-Aldrich, 700 ng/ml) for 12, 15, and 18 h. Afterwards, cells underwent the electrotransfection process using two pulses of either 250 or 300 V. For the voltage optimization, we used the pSGD62 vector. In all electrotransfection conditions, pSGD plasmids carrying gRNA-69 and gRNA-72 which had no effect on the Venus expression via NHEJ pathway were used. Nocodazole pre-treatment of cells were carried out for 15 h before the electroporation process. For comparison, 15 h post-treatment of electroporated cells with SGD constructs was also carried out.

In another experiment, a series of 0, 1, 3, 6, and 10 µl of nocodazole was included in the electroporation media which produced 0, 7, 20, 40, and 70 ng/µl of final concentration for nocodazole, and the pre-treated cells with nocodazole were electroporated with the SGD plasmids. The medium was changed 12–15 h after the electroporation, and cells were cultivated for 10 days and then used for the assessment of the Venus fluorescence.

For assessment of the RNase HII effect on the HDR rate, pre-treated cells with nocodazole (700 ng/ml) for 15 h were electrotransfected with a mixture of either of 0, 1, 5, or 10 µl RNase HII (#M0288, 5 units/µl, NEB) combined with 20 µg pSGD69/pSGD75 (2 µg/µl) and 2 million cells in 250 OptiMEM-GlutaMAX. Cells underwent two 10 ms pulses of 300 V with 10 s interval in 4 mm cuvettes. Cells were selected against puromycin antibiotic (1000 ng/ml) for 24 h and were analyzed for the Venus expression 10 days following the electroporation.

### End-point, digital, and semi-quantitative PCR

The end-point and semi-quantitative PCR was carried out using the GoTo Taq DNA polymerase kit (#9PIM300, Promega, Germany). It included 1–50 ng DNA templates, 10 µl 5× PCR buffer, 0.75 µl 10 mM dNTPs, 1 µl 10 µM forward/reverse primers, 1.5 µl 200 mM magnesium chloride, and 0.25 µl of GoTo Taq DNA polymerase in 50 µl total reaction. We used 35 cycles for end-point PCR and 33 cycles for semi-quantitative PCR. The PCR program included an initial denaturation step of 94 °C for 3 min, 35 cycles of 94 °C for 40 s, 60 °C for 30 s, and 72 °C for 60 s, followed by a 5-min extension at 72 °C.

To detect the HDR in the Venus transgene, we used two primer pairs and sent the PCR products for sequencing. To detect the HDR event in the RHA of the Venus, we used primers of Puromycin-Venus_FWD and Puromycin-Venus-REV (Table S4). The amplicon lengths were 883 bp for SGD69 and SGD77, 554 bp for SGD73 and SGD74, and 1314 bp for SGD75 groups, whereas no amplicon was expected for the wild-type Venus gene.

To quantify the copy number of Cas9 transgene in the long-term selected cells against puromycin, we used the Digital PCR technique. The sequences of primers and probes are included in Table S6. The details of digital PCR was previously described^1^. Briefly, using the IDT online software a FAM-labelled probe was designed specific for the Venus and a HEX-labelled probe was designed for the Cas9 insertion (Table S6). The Prime Time 5′ 6-FAM/ZEN/3′ IBFQ and 5′ 6-HEX/ZEN/3′ IBFQ probes were synthetized by IDT (Integrated DNA Technologies, USA), while the primer pairs were separately ordered from Eurofins Genomics (Germany). The digital PCR was carried out using the QuantStudio 3D Digital PCR device (Thermo Fisher Scientific, USA). The final concentrations of primers and probes were 250 and 900 nM, respectively. Based on the device guideline, the digital PCR reaction involved 8.7 µl of 2× master mix, 0.87 µl of 20× primer–probe assay, 1.83 µl distilled water, and 6 µl of DNA. Optimization of PCR reactions was carried out for concentration of genomic DNA (40 ng/µl). Digital PCR was carried out by the ProFlex 2× Flat PCR System with the following program: 96 °C for 10 min, followed by 39 cycles of 60 °C for 2 min and 98 °C for 30 s, followed by an extra step of incubation at 60 °C for 2 min and infinite incubation at 10 °C. The cover temperature was 70 °C, as suggested by the instructor. The emission of FAM and HEX dyes was recorded by the instrument and the data was analyzed using the online software of QuantStudio 3D AnalysisSuite (Thermo Fisher Scientific).

### Fluorescence microscope imaging and flow cytometry analysis

Cell viability was defined as cell numbers in the electroporated group divided by the cell number in the negative control with no pulsing. The efficiency of HDR- and NHEJ-induced knock-outs of the Venus reporter was assessed 10 days after the electroporation. The Hoechst staining of the cells was carried out via a 30-min incubation at 37 °C in OptiMEM (without phenol) supplemented with 1% fetal calf serum (FCS) and Hoechst 33342 (2 µg/ml final concentration). Various areas of the cell plates were randomly chosen and photographed under the fluorescence excitation. In addition, using flow cytometry by a MACSQuant Analyzer, we evaluated the number and rate of cell carrying a knock-out.

### Statistical analysis

Means comparison was carried out using the least significant difference (LSD) test, considering a p-value < 0.05 as a significant difference. All experiments were carried out at least three times.

## Results

### Establishment of a platform for detection of HDR rate in Venus transgene

**Figure 1:**
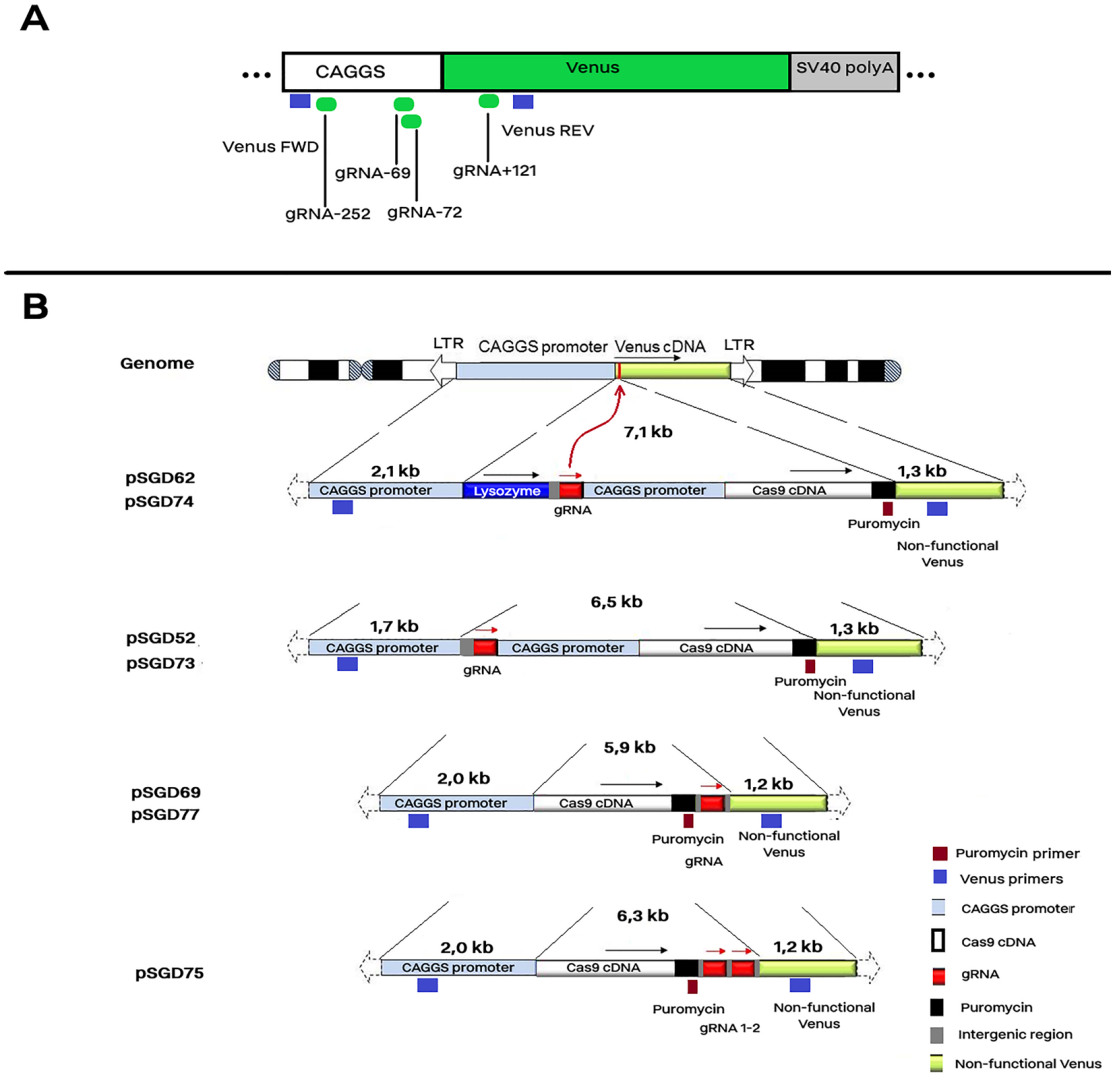
Schematic presentation of the gRNAs and large plasmids. (**A**) The location of three gRNAs on the CAGGS promoter and one gRNA on the 5´coding part of the Venus transgene. Forward and reverse primers for amplification of Venus are depicted in blue colour. (**B**) The genomic location of Venus transgene and its homology arms with SGD constructs as well as the insertion cassettes. Compared to the pSGD52, pSGD73 and pSGD74 constructs, the gRNA was translocated after the Cas9 and puromycin genes in pSGD69, pSGD75, and pSGD77. The location of a primer complementary to the puromycin sequence is depicted with a red box. This primer was used combined with the Venus Rev primer for detection of the HDR-mediated SGD insertion into the Venus transgene. pSGD 52 and 62 contained gRNA-252, pSGD73, 74, and 77 contained gRNA-72, pSGD 69 contained gRNA-69, and pSGD75 contained both gRNA-72 and + 121. The figure was created in Microsoft PowerPoint 2016.

In a previous study, we analyzed a modified pX459 plasmid carrying different gRNAs on the expression of the Venus transgene^1^. We found three gRNAs—gRNA-252, gRNA-72, and gRNA-69—targeting the promoter region of Venus transgene, where the CRISPR-mediated NHEJ did not interfere with the Venus expression^1^ (Fig. 1a). We used these gRNAs to make the pSGD constructs. This could assist us in establishing a screening platform in which only a targeted knock-in event of the DNA donor could disrupt the Venus expression and knock-out the Venus transgene (Fig. 1b). In addition, as the positive control for transfection, we used gRNA + 121 which targeted the 5' proximal region of Venus cDNA and abrogated the Venus protein via NHEJ.

**Figure 2:**
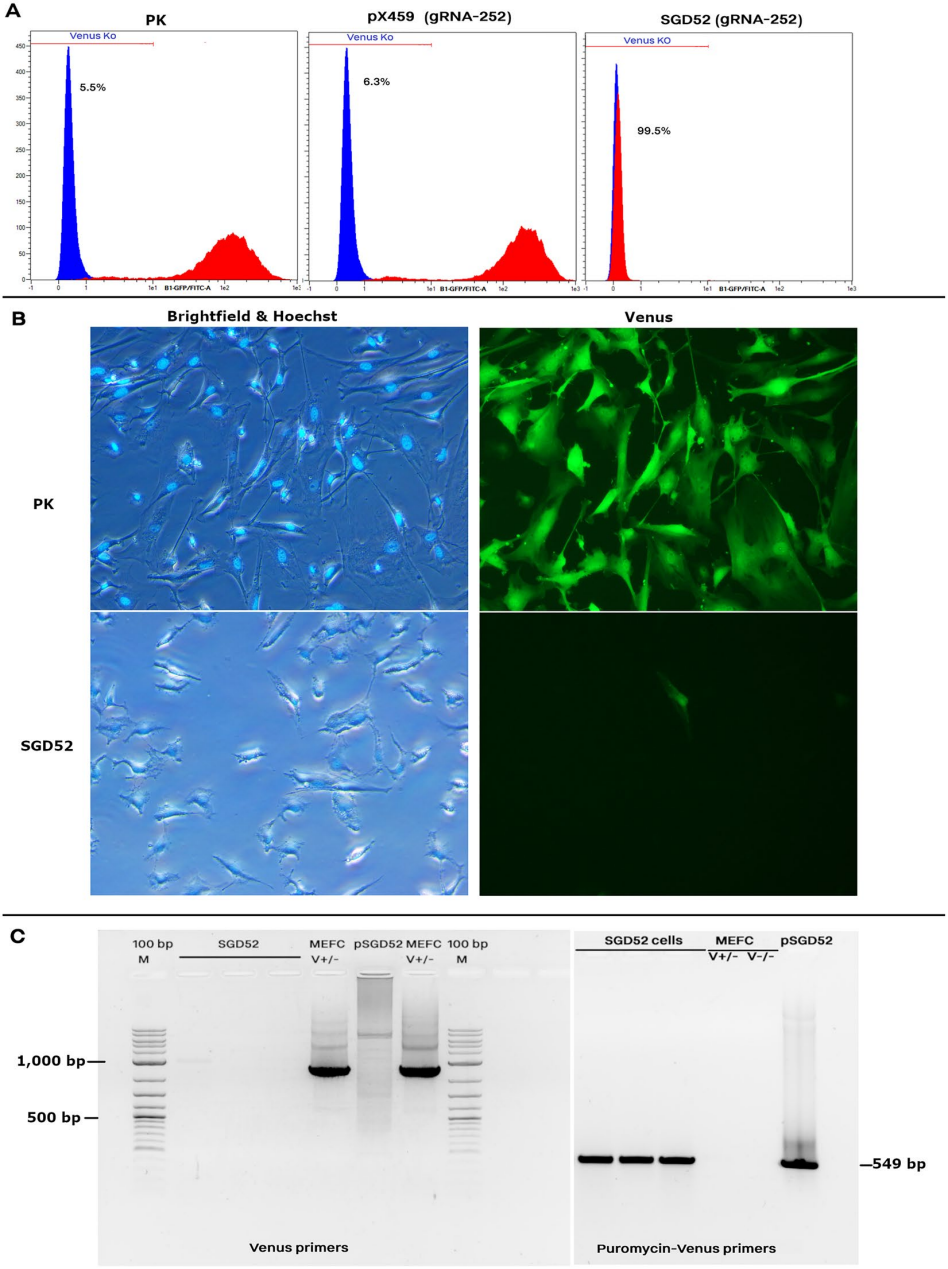
HDR-mediated Venus knock-out using the SGD52 construct. The MEF cells carrying a single copy of Venus were electrotransfected with either pX459 plasmid expressing gRNA-252 and Cas9, or the SGD52 vector expressing gRNA-252 and Cas9 and puromycin plus an HDR cassette. The pSGD52-electrotransfected cells were selected against puromycin for 105 days. In parallel, cells from the pX459-252 and the non-transfected cells (positive control), expressing a single copy of Venus, were also cultured for 105 days. (**A**) Results of FACS analysis. The Venus expression pattern of PK and pX459/ pSGD52-electroporated cells are shown in red color. The MEF cells with no Venus transgene was considered as the negative control and was depicted in blue color. (**B**) Fluorescence microscopy and (**C**) PCR. Two sets of primers were used to amplify the Venus transgene and the Puromycin-Venus. Specific primers for Venus could detect only the wild type copy of Venus transgene under the CAGGS promoter in non-transfected cells, but not the truncated Venus using the SGD cassette. The presence of the HDR-mediated SGD cassette was verified using specific primers covering the part of the puromycin transgene and the proximal part of the truncated Venus. The SGD cells are electrotransfected cells with pSGD plasmids.

In an initial experiment, MEF cells carrying one copy of Venus were electrotransfected with pSGD52 plasmid, which expressed gRNA-252, Cas9 protein, and puromycin. The electrotransfected cells were selected against puromycin for 105 days, analyzed for fluorescence expression, and assessed for the HDR event using TIDE analysis of the sequence electropherograms. Following the long-term selection, the resistant population against puromycin showed > 99% ratio of Venus knock-outs (Fig. 2a,b). Use of primer pairs specific for the Venus promoter and terminal region showed that the expected band (900 bp) was detected only in the non-electroporated group, while the SGD52 group carried only the non- functional Venus with a large inserted fragment (> 5 kb) between the Venus promoter and the terminal region (Fig. 2c). In addition, implementing primers covering the puromycin-Venus showed the presence of an expected band (549 bp) exclusively detectable in the HDR-mediated replacement of the wild type Venus with the non-functional transgene in the SGD transfected groups. The sequencing results of PCR products confirmed integration of the construct into the Venus transgene (Fig. S1). Therefore, we considered the loss of Venus fluorescence as the CRISPR/HDR-mediated knock-in event.

**Figure 3:**
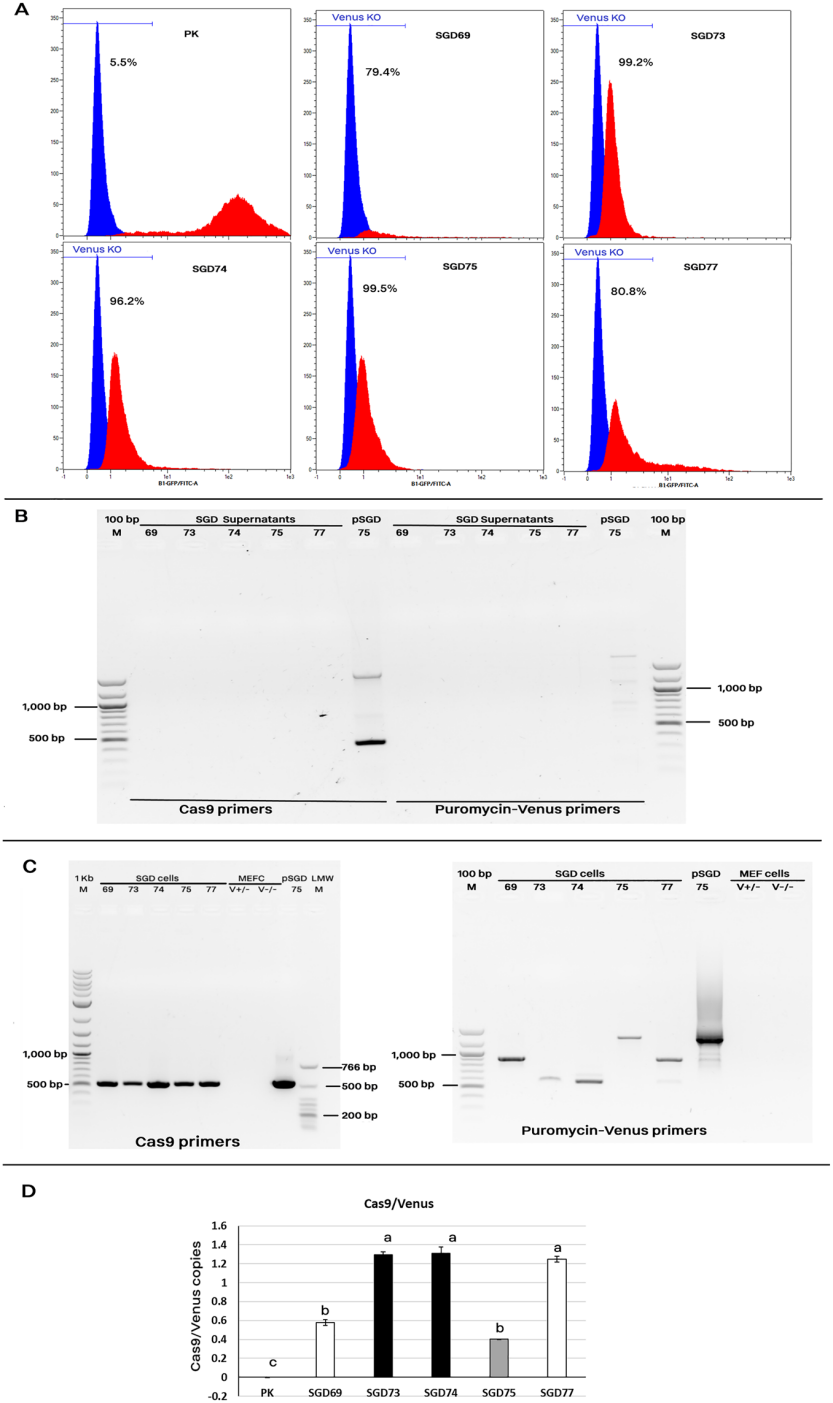
HDR-mediated Venus knock-out using various SGD constructs. The MEF cells carrying a single copy of Venus transgene were electrotransfected with pSGD plasmids. The pSGD75 plasmid which carried gRNA + 121 and induced NHEJ-mediated Venus knock-out was used as the transfection control. The electrotransfected cells were selected with puromycin for 105 days. (**A**) Results of FACS analysis. The Venus expression pattern of either PK or pSGD-electrotransfected cells are shown in red color. The MEF cells with no Venus transgene were considered as the negative control and was depicted in blue color. (**B**) and (**C**) End-point PCR using two sets of primer pairs on supernatants and genomic DNA of SGD groups, respectively. After 105 days, pSGD-electroporated cells were DNaseI-treated. A fraction of supernatants was used for PCR to assess plasmid carry-over from the initial step of electroporation. Genomic DNA was extracted from cell pellets and used for PCR. Two sets of primers were used to amplify the puromycin-Venus which was detectable only in the HDR events although with different lengths of amplicons. Moreover, Cas9 primers were used for detection of the Cas9 transgene. This primer set produced a same band of PCR product in all SGD groups. (**C**) Digital PCR assays specific for detection of Venus and Cas9 sequences. Two sets of primer–probe assays were used to detect the copy number of Venus and Cas9 genes and the rate of Cas9 copies to Venus copies was calculated.

Then, we used pSGD69 expressing gRNA-69, and pSGD73, pSGD74, and pSGD77 expressing gRNA-72 (Fig. 1b). The pSGD75 construct expressing both gRNA + 121, which efficiently induced gene knock-out via NHEJ, and the gRNA-72 was used as the transfection control. Electrotransfection of MEF cells carrying the Venus transgene with these five large plasmids was carried out and cells were selected with puromycin after the electroporation for 2 days. At day 10 following the electrotransfection, cells were screened for Venus expression. A fraction of these cells were used for sequencing. Results showed the existence of NHEJ-mediated indel mutations (Fig. S2). Because all the pSGD casssettes included puromycin, the remained cells after day 10 were cultured in a puromycin-supplemented medium for a period of 90–120 days. After 105 days of cultures, a 79–99% Venus knock-out was observed in different groups (Fig. 3a). The rate of Venus knock-out cells increased as the culture period was prolonged (Fig. S3). The majority of Venus knock-out was observed after 75 days of incubation. However, for the pSGD74 (13.4 kb), > 90 days culture of electroporated cells against puromycin was required to have a population with a high loss of Venus signal (Fig. S3). To avoid the possibility of the plasmid carryover from the early electroporation step, DNaseI treatment of cells was carried out after a 105-day culture of the electrotransfected cells. A sample of supernatant was collected from each group, and the cell pellets were used for DNA extraction. PCR results showed the presence of Cas9 transgene in the electrotransfected cells, but not in their corresponding supernatant (Fig. 3b). The puromycin-Venus conjunction, the ligation site of the inserted construct and RHA, was also confirmed in all electrotransfected groups by PCR (Fig. 3c) and sequencing (Fig. S4). The TIDE analysis of the sequenced fragments showed highly efficient HDR rate in all SGD constructs (Fig. S4). Therefore, the ability of the screening platform for detection of HDR rate in Venus transgene was reproduced using various pSGD plasmids, ranging from 12.0 to 13.4 kb. Using two digital PCR assays, a similar rate of Cas9/Venus copies was observed for in SGD73, SGD74, and SGD77 groups, ranging from 1.2 to 1.3) (Fig. 3c,d). However, the rate of Cas9/Venus copies ranged from 0.4 to 0.6 in SGD69 and SGD75 groups. These results showed different HDR efficiency of different SGD cassettes. Predicted off-target sites on the mouse genome is shown in Table S5. Since the gRNAs were designed to target an exogenous DNA in the mouse genome, a limited number of off-target sites were predicted for each gRNA.

### DNA quality is important for efficient electroporation of large constructs

**Figure 4:**
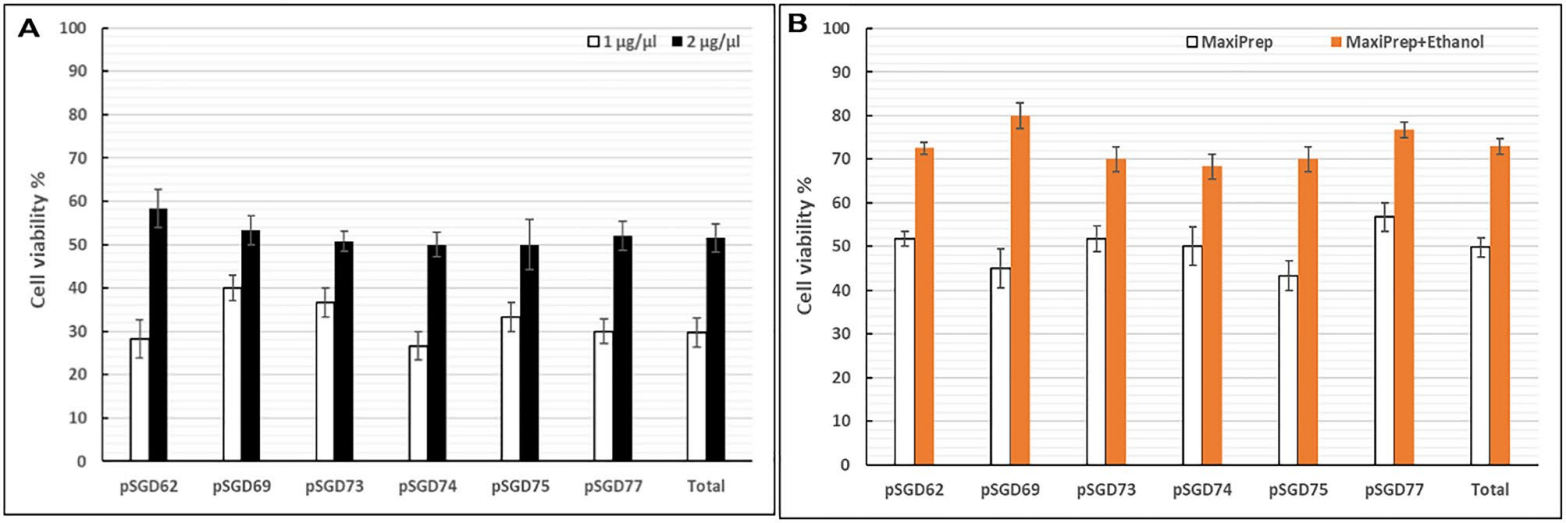
Evaluation of DNA quality on the cell survival rate following electroporation. (**A**) Twenty micrograms of SGD plasmids were used for electrotransfection either in 10 µl (2 µg/µl) or 20 µl (1 µg/µl) of the electroporation medium (250 µl). (**B**) The effect of ethanol washing of anion exchange column-prepared DNA was assessed on the cell survival rate using different large plasmids.

The effect of DNA preparation and concentration on the rate of cell survival was assessed. We used 20 µg SGD plasmids either in 10 µl (2 µg/µl) or 20 µl (1 µg/µl) in the electroporation medium (250 µl). Reducing the DNA volume significantly improved the cell survival rate, on average 20% for all SGD constructs (Fig. 4). Then, we assessed if ethanol washing of anion exchange column-prepared DNA can improve the cell survival rate. The ethanol-purified plasmids (2 µg/µl plasmid concentration) considerably increased the cell survival rate for all SGD constructs (Fig. 4). The cell survival rate was upgraded to 73%, on average for all plasmids.

### TILD constructs improved neither the HDR nor the NHEJ rate

**Figure 5:**
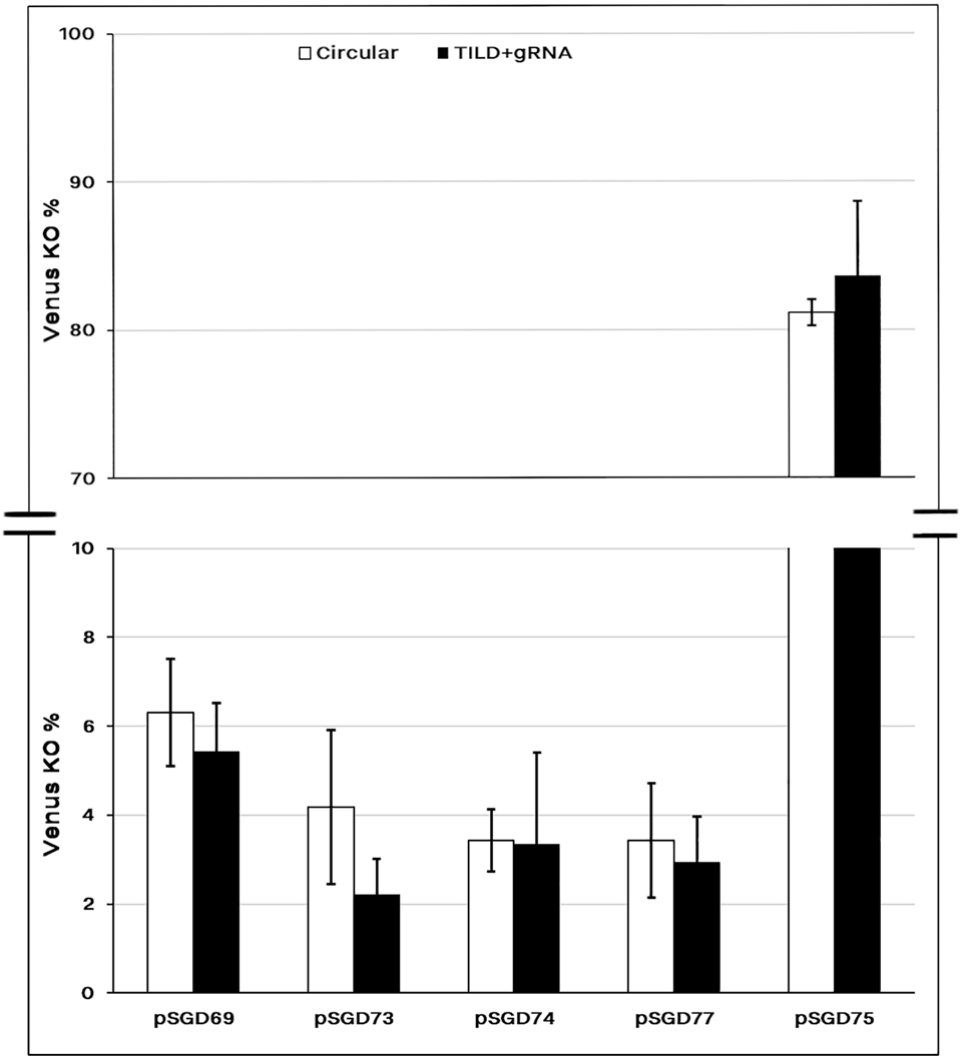
Comparison of TILD versus circular DNA donors for HDR induction. The pSGD plasmids carrying the CRISPR system and the DNA donor which had *BamH1* restriction sequence at both 5′ and 3′ ends. The constructs were digested with *BamHI* enzyme to release the TILD fragments and co-transfected with the corresponding gRNA-encoding plasmid into MEF cells carrying the Venus transgene. The Venus knockout rate was compared between TILD and circular plasmids. The same process was used for the pSGD75 which induced both HDR and NHEJ knockout. This plasmid was considered as the positive control for transfection rate.

Electrotransfection of ethanol-purified SGD constructs (2 µg/µl) induced > 82% transfection efficiency using the pSGD75 plasmid expressing gRNA + 121 which induced Venus KO via NHEJ event (Fig. 5). The SGD construct was digested with *Bam* HI to make TILD fragments. Co-electroporation of the TILD constructs with the pX459 plasmid encoding the corresponding gRNA did not increase the transfection rate either for NHEJ-induced pSGD75 construct or HDR-induced SGD constructs compared to that of circular SGD construct (Fig. 5).

### RNAse HII inclusion into the electroporation medium improved the HDR rate

**Figure 6:**
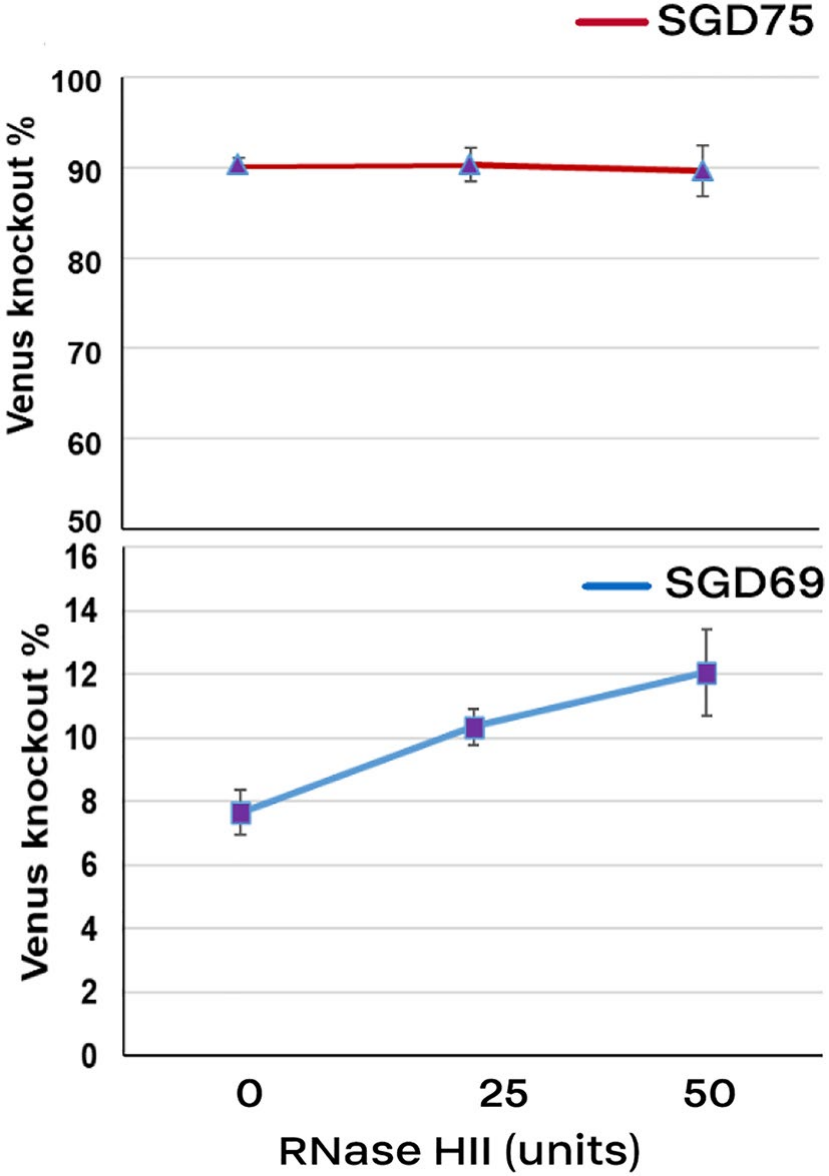
Effect of RNase HII on the HDR rate. MEF cells were electrotransfected with pSGD plasmids carrying both the CRISPR system and DNA donor. We included different concentration of RNase HII into the electroporation media. The pSGD75 plasmid caused both NHEJ- and HDR-induced Venus knock-out.

As shown in Fig. 6, cells were directly electroporated with the electroporation media involving the RNase HII enzyme. Implementation of RNase HII increased the HDR rate using pSGD69 plasmid. Higher concentration of RNase HII (10 µl or 50 units per reaction) had a stronger effect on the HDR rate (12%). On the other side, the NHEJ rate which was assessed by using pSGD75 plasmid was not affected by RNase HII.

### The effect of cell pretreatment with nocodazole on the HDR rate

**Figure 7:**
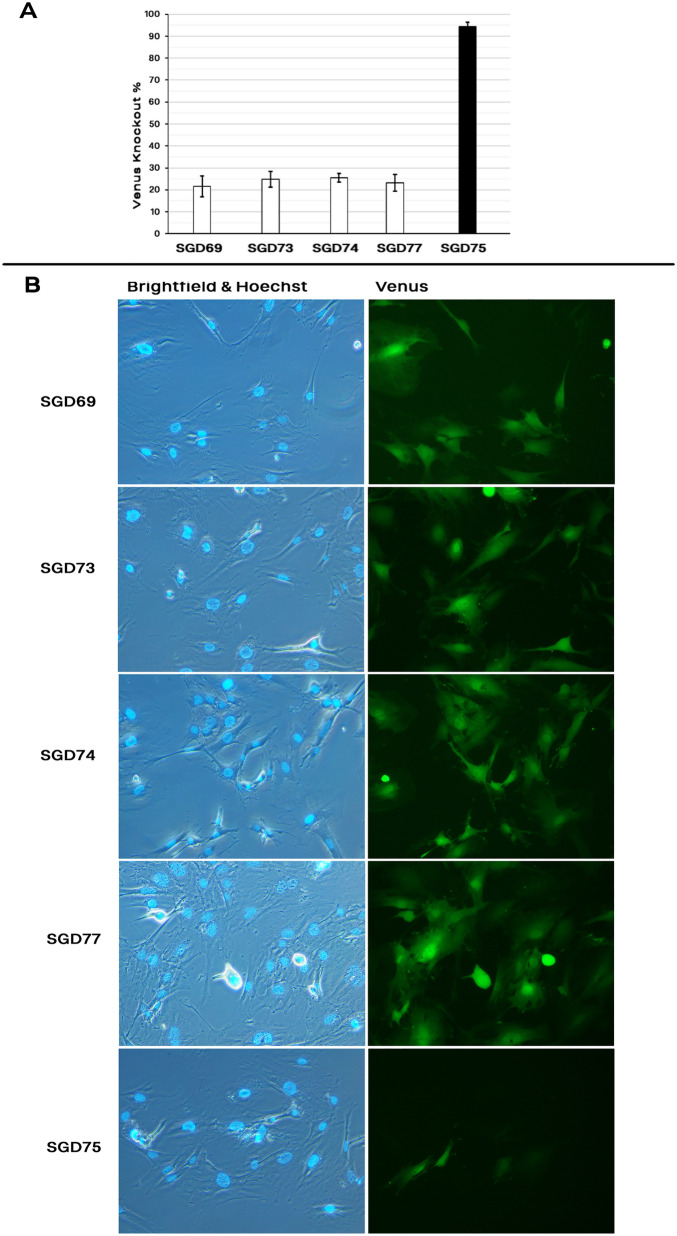
**E**ffect of nocodazole on the HDR rate. MEF cells were electrotransfected with pSGD plasmids carrying both the CRISPR system and DNA donor in the presence of nocodazole into the electroporation media (70 ng/µl final concentration). The pSGD75 plasmid associated with both NHEJ- and HDR-induced Venus knock-out. (**A**) the HDR-mediated Venus knock-out rate using SGD cassettes. (**B**) Fluorescence microscopy of MEF cells electroporated with the pSGD plasmids.

We pretreated MEF cells with 700 ng/ml nocodazole and electroporated them with two pulses of 300 V (Fig. S5). The majority of cells were killed following the optimized protocol. Then, we optimized the pretreatment time as well as the voltage pulsing. Irrespective of the treatment time, nocodazole-pretreated cells were very sensitive to 300 V. However, they could resist two pulses of 250 V with more than 80% viability following a 15-h pretreatment time (Fig. S5). Prolonging the nocodazole incubation time to 18 h decreased cell viability following conduction of both 250 and 300 V (p-value < 0.05). Puromycin selection of the transfected cells reduced the cell viability in both 12 and 18 h pretreatment groups. However, the 15 h-pretreatment group maintained the high rate of cell viability after the puromycin selection in both 250 and 300 V. This indicated that a highly efficient transfection was achieved via conducting the 250 V, while maintaining the cell survival rate. The rate of Venus knock-out was assessed in the 15 h-pretreated cells with twice pulsing of 250 V (Fig. S6). Nocodazole pretreatment slightly improved the HDR rate for pSGD69 and pSGD77. However, the pretreatment did not affect the rate of NHEJ-induced knock-out using the pSGD75 plasmid (Fig. 7, p-value > 0.05).

### Nocodazole inclusion into the electroporation medium considerably increased the HDR rate

A series of 0, 7, 20, 40, and 70 ng/µl of final concentration for nocodazole was supplemented into the OptiMEM-GlutaMAX medium containing the pSGD69 vector (Fig. S7). To avoid the side effect of nocodazole on the cell viability and proliferation, the culture medium was changed 15–18 h after the electroporation (Fig. S8). Assessment of cell viability at 24 h after the electroporation showed that higher rate of cell viability using higher amount of nocodazole. Selection of electroporated cells against puromycin also confirmed the higher cell survival using 70 ng/ µl of nocodazole into the electroporation medium. After a 1-day selection against puromycin, cells were cultured with no antibiotic selection for an 8-day period. Then, a fraction of cells was analyzed for Venus fluorescence using FACS analysis. Using the pSGD69 plasmid, inclusion of 0–40 ng/µl nocodazole into the electroporation medium did not significantly improved the HDR rate (p-value > 0.05). However, inclusion of 70 ng/µl nocodazole into the electroporation medium could considerably improve the Venus knock-out rate (21%) compared to both control and sham groups (3% DMSO) (p-value < 0.05). Then, all five plasmids were co-electroporated with 70 ng/µl nocodazole into the MEF cells. FACS results of Venus knock-out rate at 10 days after the electroporation reproduced high rate of Venus knock-out using pSGD69, expressing gRNA-69, and pSGD73, 74, and 77 expressing gRNA-72 (21–26%). Electroporation of the pSGD75, expressing both gRNA-72 and gRNA + 121, caused 95% Venus knock-out.

## Discussion

We’ve recently optimized the plasmid-based CRISPR technology to induce error-prone NHEJ and targeted deletion in mouse fibroblast cells^1^. On-target knock-in of large constructs into mammalian genome has been a challenging issue using the CRISPR/Cas9 technology^5^. In the current study, we were interested in implementing this technique for the knock-in of large constructs, comprising 5.3–7.1 kb insertion length and 3.0 -3.5 kb homology arms. We used all-in-one plasmids ranging from 12.0 to 13.4 kb as an integrated cassette including the CRISPR/Cas9-expressing sequence and the DNA donor. Basically, electroporation of large plasmids is inefficient and associated with a high rate of cell mortality in primary cells^8^. In the current study, a transgenic fibroblast line harboring a single copy of Venus transgene was subjected for targeted knock-in events. Three guide RNAs which targeted the Venus promoter and did not induce an NHEJ-interruption of the Venus expression were used to make the SGD constructs^1^. Therefore, unless a targeted knock-in occurred, no Venus knock-out was expected using the SGD constructs. This provided us a direct detection platform, apart from the so far used qPCR assay^23,24^. The electrotransfection was carried out in an OptiMEM-GlutaMAX medium, which has been shown to be compatible for primary cells^1,25,26^.

Electrotransfection of MEF cells with the pSGD52 plasmid, expressing gRNA-252, lysozyme transgene, Cas9 protein, and puromycin, was carried out in an OptiMEM-GlutaMAX medium. Long-term selection of the electrotransfected cells against puromycin showed that almost all of the resistant cells did not express the Venus fluorophore. The HDR-induced knock-in event was confirmed by PCR and digital PCR as well as the electropherogram analysis using the online TIDE software. This provided molecular evidences supporting the idea that use of these gRNAs assisted establishing a screening platform in which only the CRISPR/HDR-mediated knock-in event of the DNA donor could disrupt the Venus expression. Then, we used a set of four SGD constructs with different lengths of homology arms and insertion cassettes as well as variable gRNAs inducing HDR-mediated knock-in of SGD constructs. Moreover, a pSGD cassette carrying a gRNA targeting the beginning part of the Venus transgene was used as the electrotransfection positive control by which the Venus knock-out could also be mediated through NHEJ events^1^. Similarly, long-term selection of electrotransfected cells with the pSGD plasmid set led to production of puromycin-resistant cells with no or negligible Venus expression. A difference was observed in the rate of Venus knock-out at different time periods following the electroporation. More importantly, the majority of MEF cells which were transfected with pSGD74, the largest size (14.3 kb), remained Venus-expressing while being resistant against puromycin for > 65 days. A similar trend was evident for other pSGD plasmids. This could be likely due to the presence of an episomal state for large plasmids throughout 20–25 passages. Therefore, using qPCR for estimation of the HDR rate should be avoided at early stages following the electroporation of large constructs^23,24^. We assumed larger plasmid might remain for a long time at the episomal state. However, prolonging the incubation time to 105 days considerably increased the rate of resistant cells without Venus expression (80–99%). The HDR events were confirmed by PCR, digital PCR, and TIDE analysis. Therefore, the HDR screening platform was reproduced with other SGD cassettes. Results of digital PCR showed a similar rate of Cas9/Venus copies in SGD73, SGD74, and SGD77 groups. However, for SGD69 and SGD75 cassettes, we found that the Cas9 copy number was almost half of the Venus copy number. This indicates that apart from the full-length integration, partial integration was also directed in some HDR events. Partial integration of DNA donors have been reported using both zinc-finger nuclease^27^, and CRISPR/Cas9 approaches^28–30^. We also predicted off-target effects of the CRISPR/Cas9 system using an online webtool. Because the targeted sequence was an exogenous DNA per se, there was limited number of off-target sequences, 1–2 off-target per each gRNA, for the gRNAs in the mouse genome. Although the off-target rate was not assessed in the current study, the presence of mismatches in positions 2 or 4 from the PAM sequence as well as simultaneous existence of three mismatches do not indicate high rate of off-target events for the gRNAs^31^. However, long-term presence of long plasmids expressing the CRISPR/Cas9 system in the transfected cells might exacerbate the off-target rate^31^.

One of the main obstacles for efficient electrotransfection of large plasmids is the high rate of cell death^8^. Our results for electroporation of SGD constructs using the OptiMEM-GlutaMAX medium and twenty micrograms of plasmids also confirmed low survival rate after the electroporation. Use of two-fold higher concentrations of the large plasmids could considerably upgrade both cell survival and transfection rate. Additionally, ethanol washing of the eluted DNA from the Maxi-Prep kit advocated the cell survival after the electrotransfection. Although we used toxin binders for the column purification process, the extracted plasmids might include important nuisance factors and remnant toxins from the bacterial culture^32^. Concentrating the plasmids could reduce the required volume of DNA in the electroporation media and subsequently improve the conductivity parameters for efficient electroporation and cell survival^33^. Using high quality DNA, highly concentrated and ethanol-purified plasmids, resulted in Venus knock-out rates of > 80% with a large plasmid encoding gRNA + 121. This indicated that apart from the high rate of cell survival, the electrotransfection protocol was highly efficient for gene knock-out based on the NHEJ approach. Then, we focused on improving the HDR rate of large plasmids. We compared the DNA donors in the circular and linearized forms. The HDR rate ranged from 4 to 8% for different SGD plasmids. Although supplementing DNA donor in the linearized form, considered as the TILD method, has been reported to be effective for short fragments^3,34^, we detected no advantage in using TILD fragments for large DNA donors. We need to consider that the original TILD-CRISPR approach was carried out mainly with preformed RNP complexes (gRNA and Cas9 protein) in mammalian zygotes, while in in this study, the insertion cassettes included a plasmid-based CRISPR/Cas9 system. Moreover, only for small constructs, ranging from 0.6 to 2.3 kb, the TILD-CRISPR approach produced a higher rate of HDR compared to other types of plasmid donors, while for the 6 kb insertion cassette the HDR rate was similar to the current study (6.9%)^3^. It has been shown that double-stranded DNAs rather than single-stranded DNA donors reduced the HDR efficiency drastically^11^. The current study showed that plasmids can efficiently been used as double strand DNA donors for large insertion cassettes.

Cell synchronization has been considered as an effective approach to improve the HDR efficiency. Nocodazole pretreatment of cells improved the HDR using both single strand and double strand DNAs in various cell types^10,11^. In the current study, the duration of nocodazole pretreatment and the strength of the electric pulses were important factors for cell survival after the electrotransfection. Although nocodazole pretreatment did not change the rate of NHEJ, it was relatively effective for HDR rate using two plasmids. Then, we included the nocodazole into the electroporation cuvette. In an optimized protocol, we concluded that inclusion of 70 ng/µl nocodazole into the electroporation media had the highest rate of HDR rate. The procedure was reproduced using four large plasmids. On average, using 70 ng/µl nocodazole concentration could induce a HDR rate of 23.5% for all plasmids. This was almost four-fold higher than the electroporation media without nocodazole. For small constructs, using double strand than single strand DNA with the same sequence of homology arms and integration sequence was unfavored for the HDR rate^11^. Also, knock-in of large single stranded oligonucleotides, 2762 nucleotides length, created a 7% on-target knock-in using RNP microinjection in mouse zygotes^35^. Efficient transfection of mammalian cells for knock-in of large constructs was restricted to < 3 kb insertion cassettes using the CRISPR-mediated HDR approach^5,24^. However, our results showed the high efficiency of double strand DNA donors to mediate HDR events. To our knowledge, this is the highest HDR rate which has been reported using the CRISPR technology. This study clearly showed that immediate increase of the nocodazole concentration in cells via electroporation is a robust approach for inducing the HDR rate.

In the current study, we used RNase HII as an alternative method to mediate HDR event. We used *E. coli*-derived RNase HII, which is a monomeric 21.5 kDa protein^36^. Using the electroporation method, RNase HII was delivered into the MEF cells concomitantly with the pSGD plasmid. We detected a positive trend in the HDR rate by increasing the RNase HII concentration. Moreover, unlike nocodazole-electroporated cells, electroporated cells with RNase HII did not showed multi-nuclei feature and no halt of cell division. This indicates that RNase HII is not a delaying factor for cell division, while nocodazole has been confirmed as the blocking agent for the cell cycle at G2-M phase^10,11^. Nonetheless, RNase HII function on DSBs created by the CRISPR/Cas system is yet to be explored.

The majority of findings on the action mechanism of RNase HII in DNA repair is restricted to yeasts. Knock-out of RNAse H in *Saccharomyces cerevisiae* considerably increased their sensitivity to environmental stress^15^. Similarly, inducing DSBs in the RNase H depleted yeasts was not tolerated, suggesting the importance of RNase H in HR-mediated DSB repair^16^. Assessing the NHEJ- and HR-mediated DSB repair showed that deletion of Rad52 and Rad51, two main components for HR-mediated repair, drastically reduced the cell survival rate, while deletion of Pku80 (*S. pombe* KU80 homolog), an essential component for NHEJ repair, did not reduce the cell survival rate^16^. Accumulation of RNA–DNA hybrids in the RNase H depleted cells was considered the most important obstacle for the HR-mediated DSB repair^16^. DNA-RNA hybrids that form at DNA breaks should be removed to allow recombinational repair^37^. It has been shown that the same scenario apply for LINE-1 retrotransposition and lncRNAs effects on gene expression via removal of the RNAs from RNA–DNA hybrids by RNase HII^17,18^. It has been evident that CRISPR/Cas9 interacts stably with the target DNA for more than 26 h^38^. RNase HII can remove the gRNA in RNA–DNA base pairing and release this Cas9/gRNA-target DNA binding. We speculate that this strategy could partly expedite the HDR-mediated DSB repair using the combined CRISPR/Cas9 and RNase HII system.

Additionally, RNase HII interacts with several Rad molecules, is involved in DNA repair, replication, translation, and recombination^39^. Highlighting the importance of homologous recombination for cell survival, deletion of RAD51 and RNase H increased their DSBs sensitivity, while combined RAD52 deletion and RNase H deficiency is lethal for cells^39,40^. Moreover, RNase H co-immunoprecipitation by four independent antibodies showed that the contained RNA polymerase Pol I and Pol II are involved in global transcription^41^. RNase HII depletion significantly decreased the global transcription, whereas RNase HII overexpression increased the global transcription^41^. Therefore, it is also likely that RNase HII exacerbated the expression of Cas9 protein in the pSGD plasmids and mediated the HDR events.

Because RNase HII degrades RNAs involved in the DNA-RNA hybrid during replication and transcription^42^, we were interested to know if a reduction in the NHEJ-induced knock-out rate occurs via RNase HII effect. However, combination of RNase HII and a knock-out-inducing gRNA in the pSGD75 construct showed that the Venus knock-out rate was not affected by the RNase HII concentration. The TIDE analysis of the long-term selected cells showed a similar rate of HDR and indel rate in the pSGD75-electroporated cells compared to other pSGD constructs which only mediated Venus knock-outs by HDR. However, because of the transient presence of large plasmids into the electrotransfected cells, discrimination of the NHEJ- from HDR-induced knock-out rate was not possible by chromograph analysis of PCR products 10 days following the electroporation. Using electroporation in the current study could deliver bulk amounts of RNase HII, which might remain even for the next division cycle in MEF cells. Cells can tolerate RNase HII activity even during S phase^14^. Therefore, we cannot rule out that RNase HII-mediated HDR of largely available constructs in successive rounds of cell cycles.

## Conclusions

We established a detection platform to discriminate HDR from NHEJ events in murine fibroblast cells. We found that the quality of large plasmids, all-in-one vectors to induce HDR, is critical to having a high rate of cell survival and transfection. Direct delivery of nocodazole and RNase HII through the electrotransfection process rather than cell pretreatment could considerably upgrade the HDR rate of large cassettes. This devised method is very promising for efficient on-target knock-in of large constructs in mammalian genome. However, on-target knock-in of large constructs using this technology is yet to be evaluated for endogenous target sequences, other types of primary cells, as well as RNPs.

## Supplementary Information

Supplementary Information.

## Data Availability

The datasets used and/or analyzed during the current study are available from the corresponding author.
