# Supplementary material for: CRISPR/Cas9-mediated targeted knock-in of large constructs using nocodazole and RNase HII
Source: Sci Rep. 2023 Feb 15;13:2690. doi: 10.1038/s41598-023-29789-1 (PMC9931768; doi:10.1038/s41598-023-29789-1)
Supplement: Supplementary file 1 — Supplementary Information. [file 41598_2023_29789_MOESM1_ESM.docx]

**CRISPR/Cas9-mediated targeted knock-in of large constructs using nocodazole and RNase HII**

Shahin Eghbalsaied^1,2^ and Wilfried A. Kues^1*^

^1^Institute of Farm Animal Genetics, Friedrich-Loeffler-Institut (FLI), Mariensee, Germany.
^2^Department of Animal Science, Isfahan (Khorasgan) branch, Islamic Azad University, Iran.

**^*^Corresponding author:**

Prof. Dr. Wilfried A. Kues

Friedrich-Loeffler-Institut (FLI), Federal Research Institute for Animal Health,

Institute of Farm Animal Genetics

Biotechnology/Stem Cell Physiology

Höltystr. 10

31535 Neustadt, Germany

Wilfried.kues@fli.de

**Running title:** HDR of large constructs


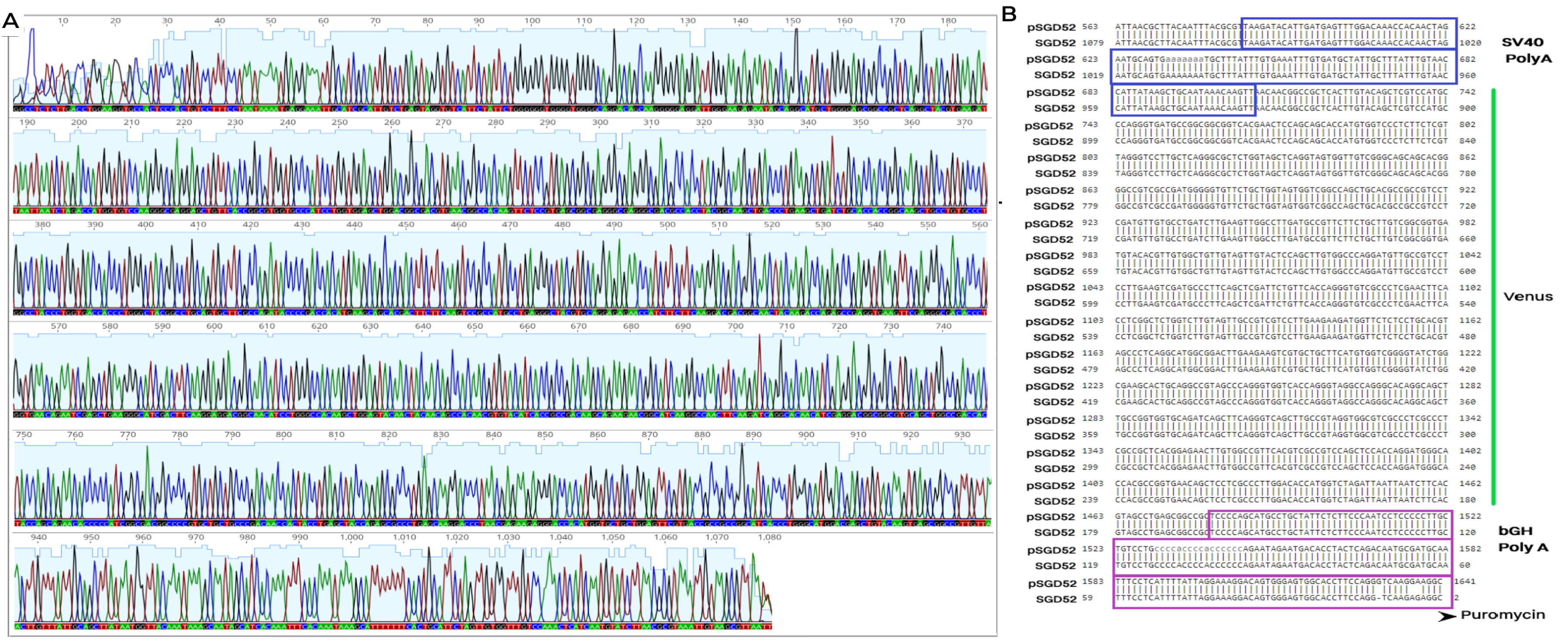


**Figure S1. Sequencing results indicating HDR-mediated Venus knockout using the pSGD52 construct.** The MEF cells carrying a single copy of Venus transgene were electrotransfected with the pSGD52 plasmid which carried gRNA-252, Cas9, and puromycin. A PCR was carried out using the Cas9-Venus primers to amplify the Cas9-Venus ligand which was detectable only in HDR events. A) the sequencing results of the PCR product. B) The pair-wise alignment of pSGD52 plasmid and the sequenced amplicon from the SGD52 transfected cells. This amplicon included the puromycin sequence followed by bGH polyA and the Venus segment which existed in the genomic DNA.


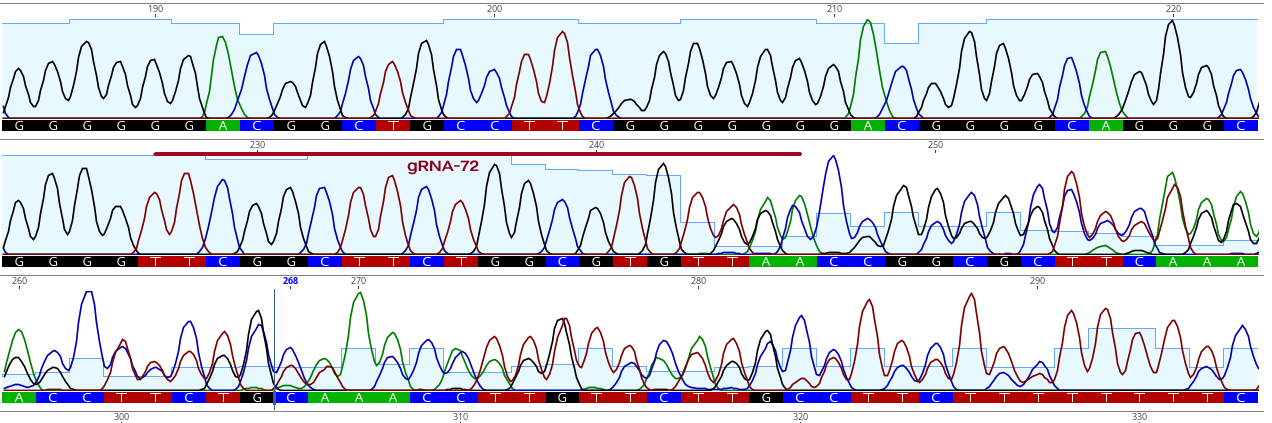


**Figure S2. NHEJ-mediated indels in the Venus promoter region that was targeted by gRNA-72 in MEF cells.** The MEF cells carrying a single copy of Venus transgene were electrotransfected with the pSGD73 plasmid which expressed gRNA-72, Cas9 protein and puromycin. At day 10 following the electrotransfection, a PCR was carried out to amplify the Venus promoter and the PCR amplicon was sequenced. Results showed the NHEJ-mediated indels in the expected region of gRNA-complementary site.


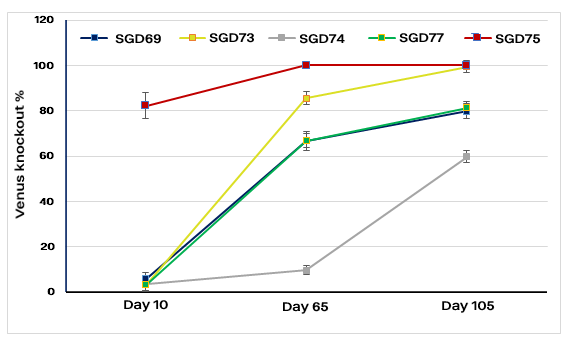


**Figure S3. The trend of Venus knockout rate using SGD constructs.** The MEF cells carrying a single copy of Venus transgene were electrotransfected with pSGD plasmids which carried either of gRNA-72 and gRNA-69, Cas9, and puromycin. The pSGD75 plasmid which carried gRNA+121 and induced NHEJ-mediated Venus knockout was used as the transfection control. The electrotransfected cells were selected against puromycin for 100 days and the Venus knockout rate was calculated at different time periods using either fluorescent microscopy or the flowcytometry analysis.


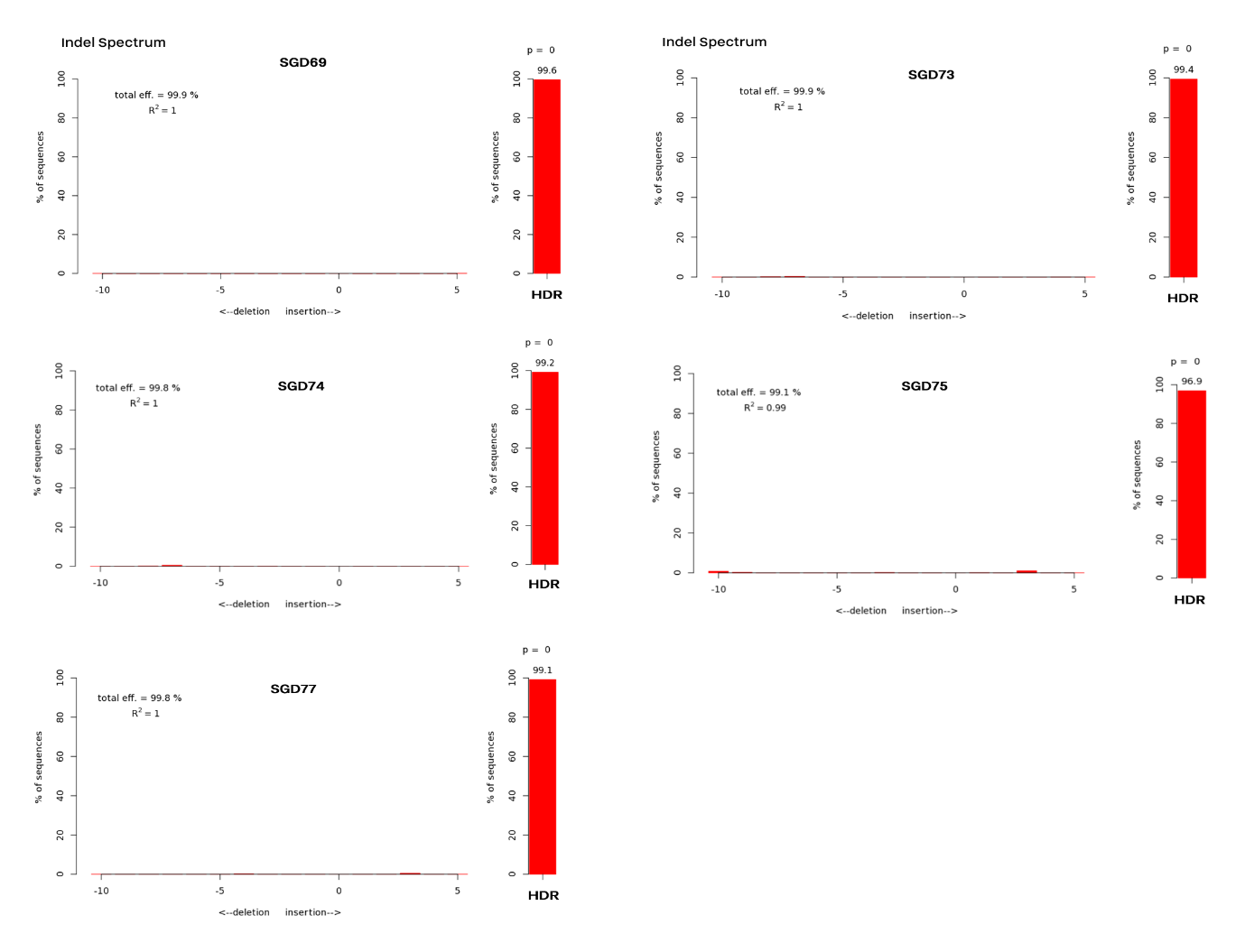


**Figure S4. Indels spectrum and HDR analysis of SGD-electrotransfected cells.** The MEF cells carrying a single copy of Venus transgene were electrotransfected with pSGD plasmids which carried the CRISPR/Cas9 system and puromycin. The electrotransfected cells were selected against puromycin for 100 days and were used for amplification of the Cas9-Venus ligand. To estimate the rate of HDR and NHEJ in the amplified amplicons, the sequencing results of PCR products were analysed using the TIDER online software (http://shinyapps.datacurators.nl/tider/) .


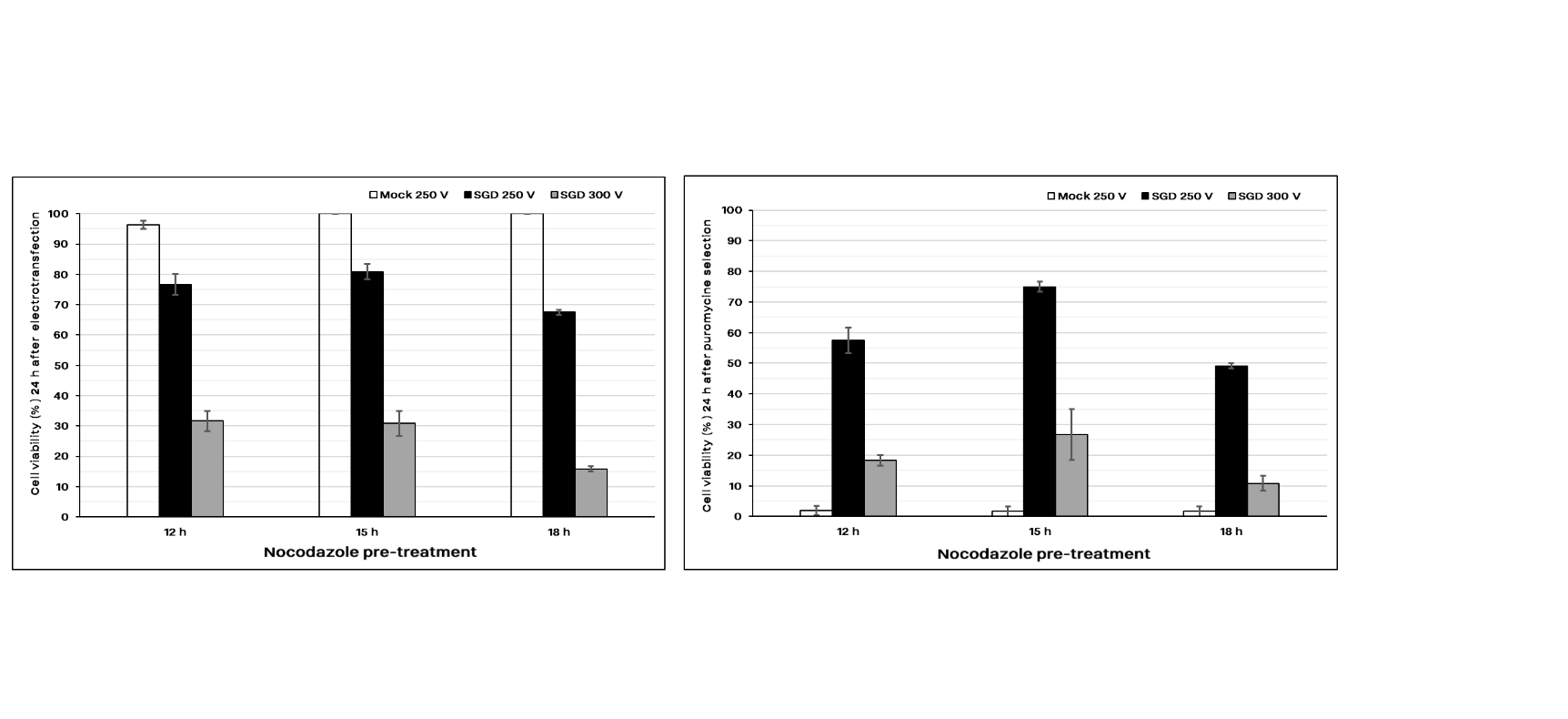


**Figure S5. Effect of nocodazole pretreatment on the cell survival rate.** Mouse EF cells were pretreated with 700 ng/µl for 12, 15, and 18 hours. Then, an electrotransfection was carried out using the pSGD69 plasmid at 250 and 300 volts, tow times pulses of 10 ms with 10 s interval. The electrotransfected cells were selected against puromycin 24 h after the electroporation. The cell survival rate was measured at day 1 (24 h after electroporation) and day 2 (24 h after puromycin selection).


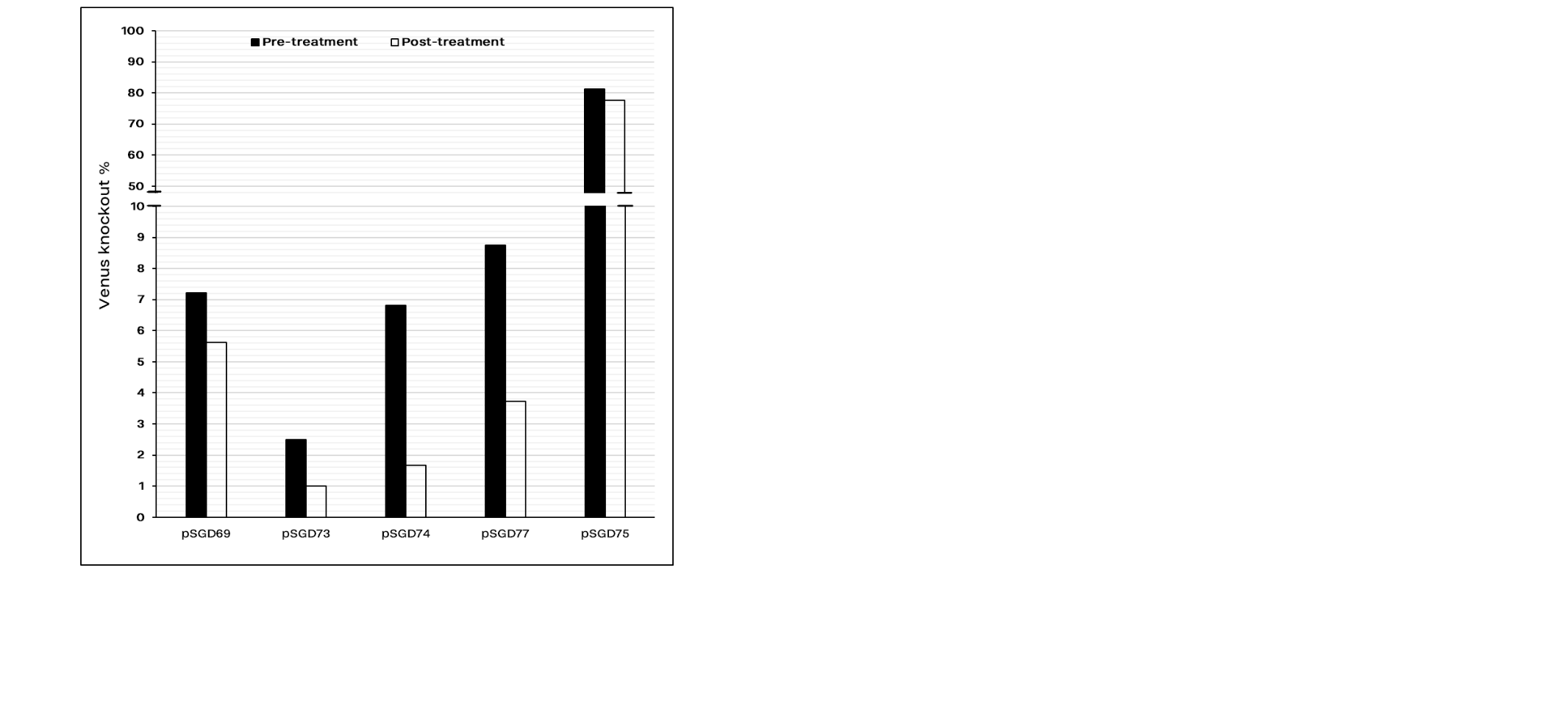


**Figure S6. Venus knockout rate in nocodazole-pre-treated and -post-treated cells.** Cells were nocodazole-treated either before or after the electroporation with different SGD constructs. The electroporated cells were selected against puromycin 24 h after the electroporation, and the rate of Venus knockout was analysed 10 days after the electroporation.


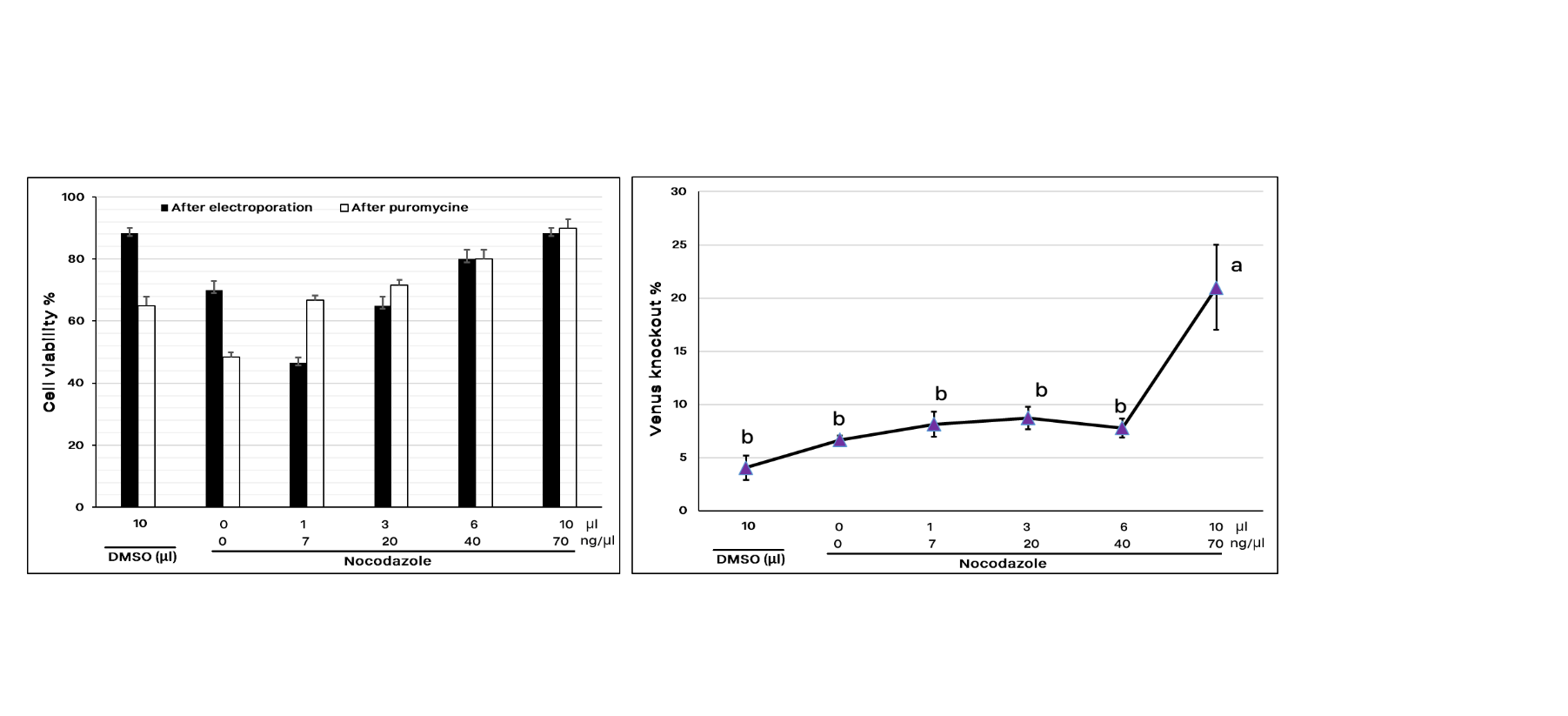


**Figure S7. Inclusion of nocodazole into the electroporation medium.** Mouse EF cells were electrotransfected with the pSGD69 plasmid carrying both the CRISPR system and DNA donor. We included different concentration of nocodazole (0, 1, 3, 6, and 10 µl) into 250 µl of the electroporation media. Cell viability was measured 24 h after the electrotransfection. The rate of HDR-mediated Venus knockout was assessed 10 days after the electrotransfection.


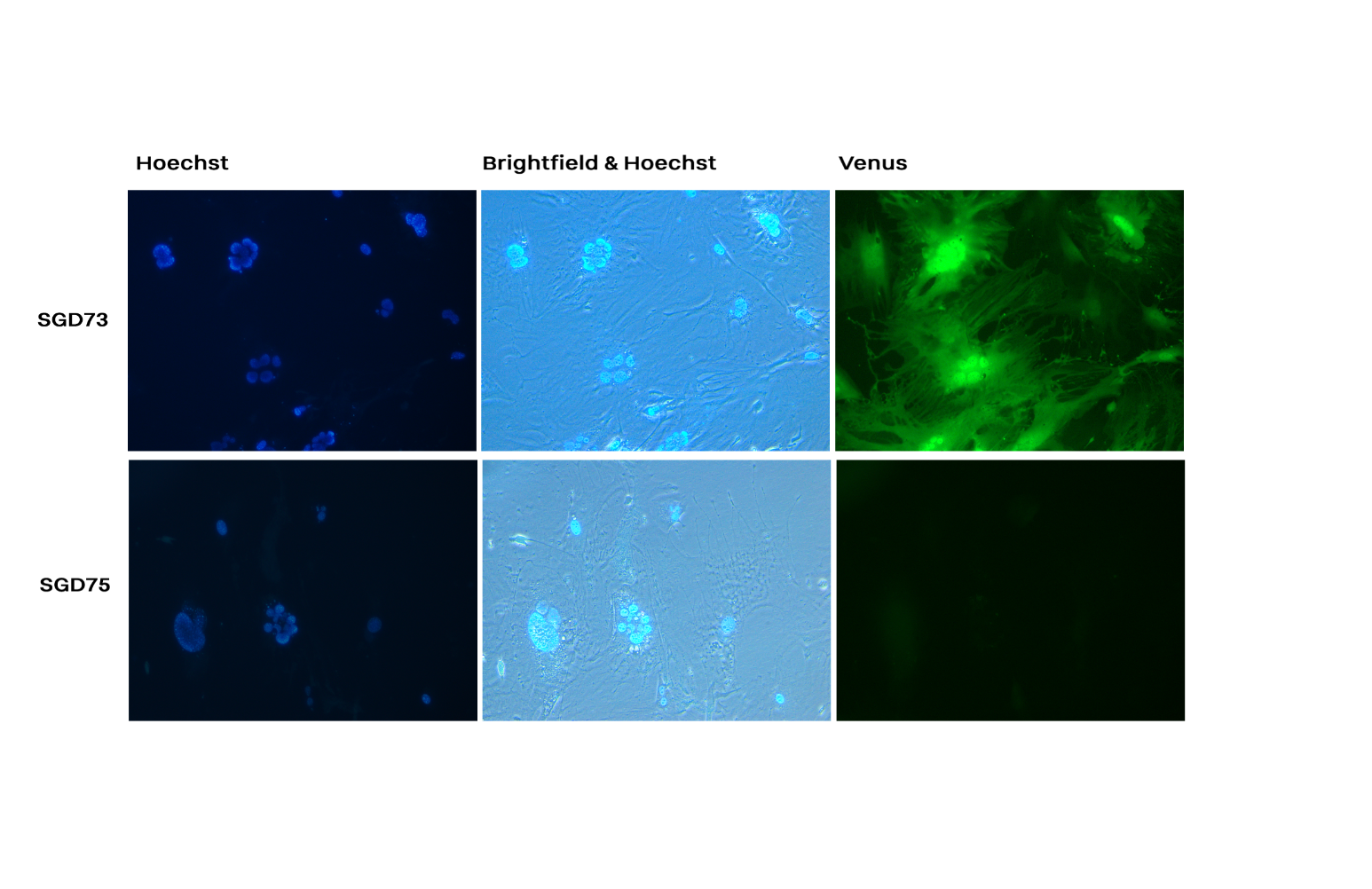


**Figure S8. The presence of multi-nuclei when nocodazole remained in the medium.** Mouse EF cells were electrotransfected with pSGD plasmids carrying both the CRISPR system and DNA donor in the nocodazole-supplemented electroporation medium (3.5 %). After the electrotransfection, nocodazole remained in the culture medium for 2 days. The nocodazole removal was required 12-18 h after the electrotransfection.


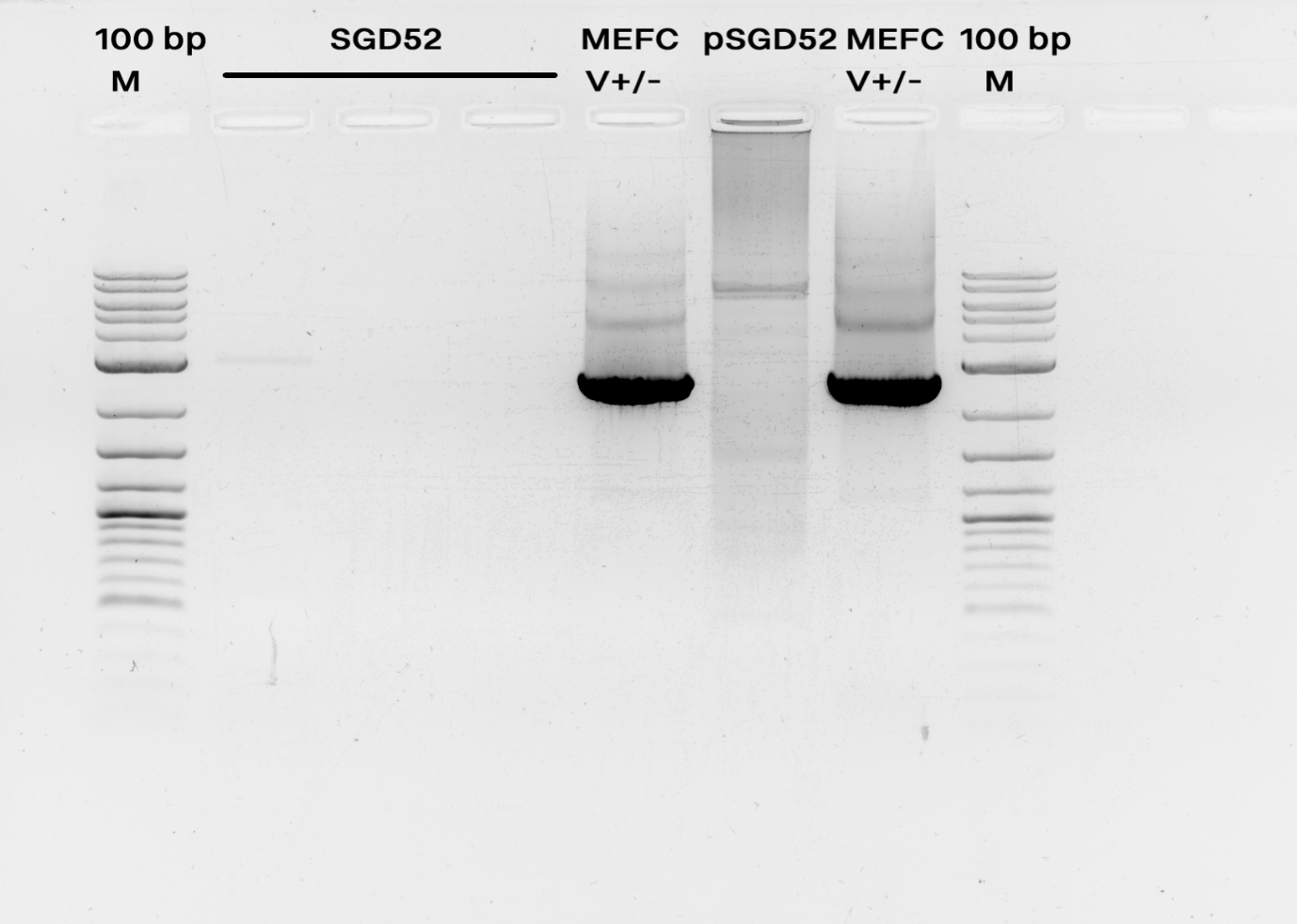


**Figure S9. Full image from a 1% agarose gel electrophoresis pertained to the left image in Figure 2C.** This image was cropped before originally, as the rest of wells were not loaded.


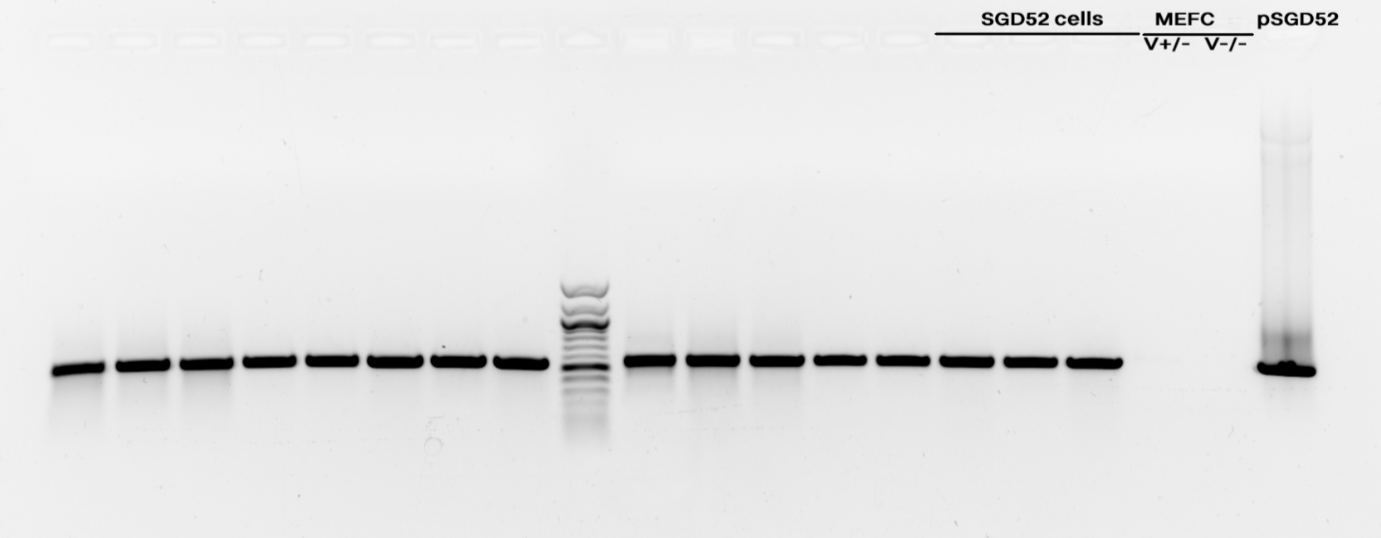


**Figure S10. Full image from a 1% agarose gel electrophoresis pertained to the right image in Figure 2C.** The rest bands in the left are PCR products from SGD52 group with different replicates and repetitions.


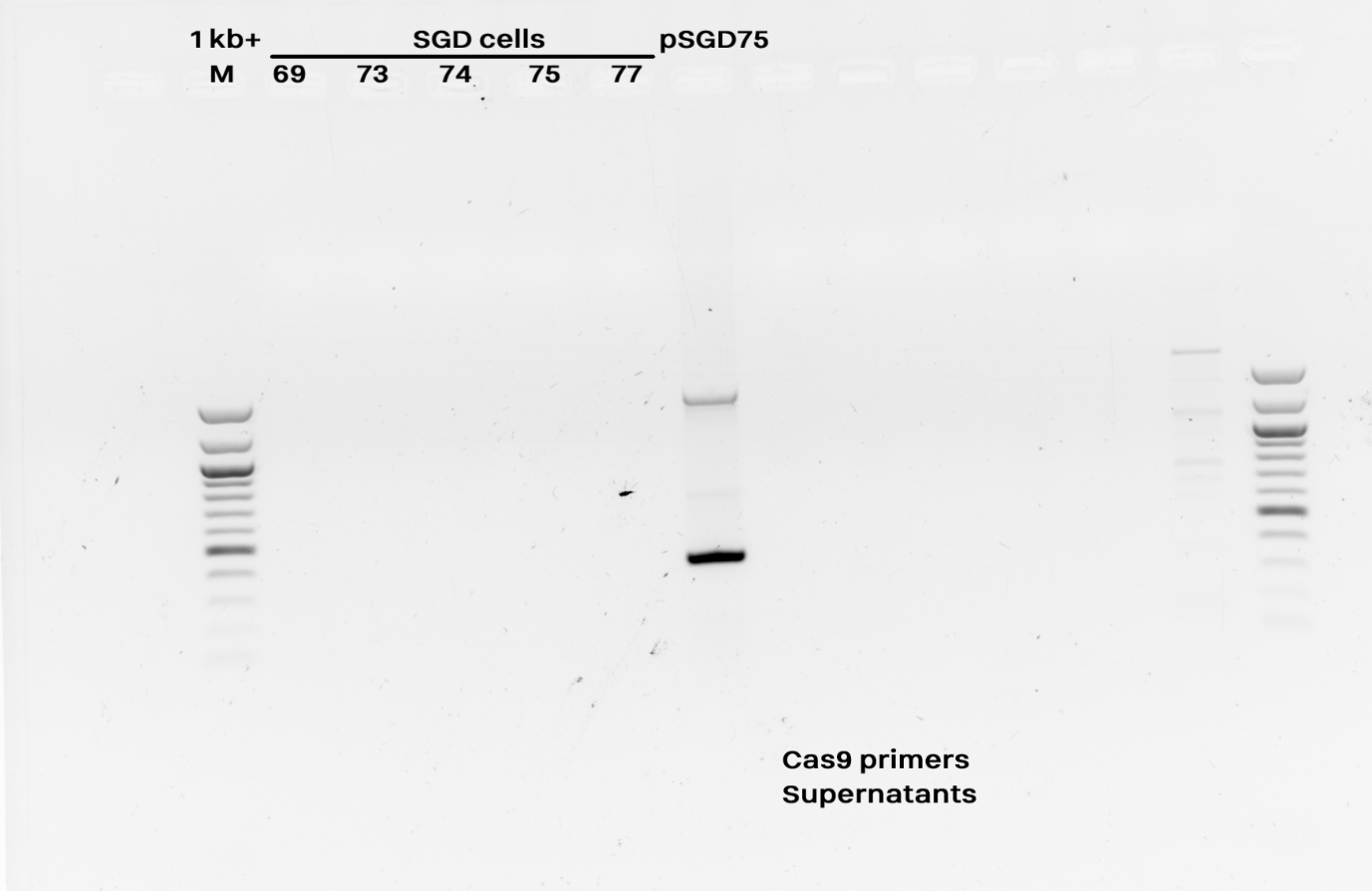


Figure S11. **Full image from a 1% agarose gel electrophoresis pertained to Figure 3B.**


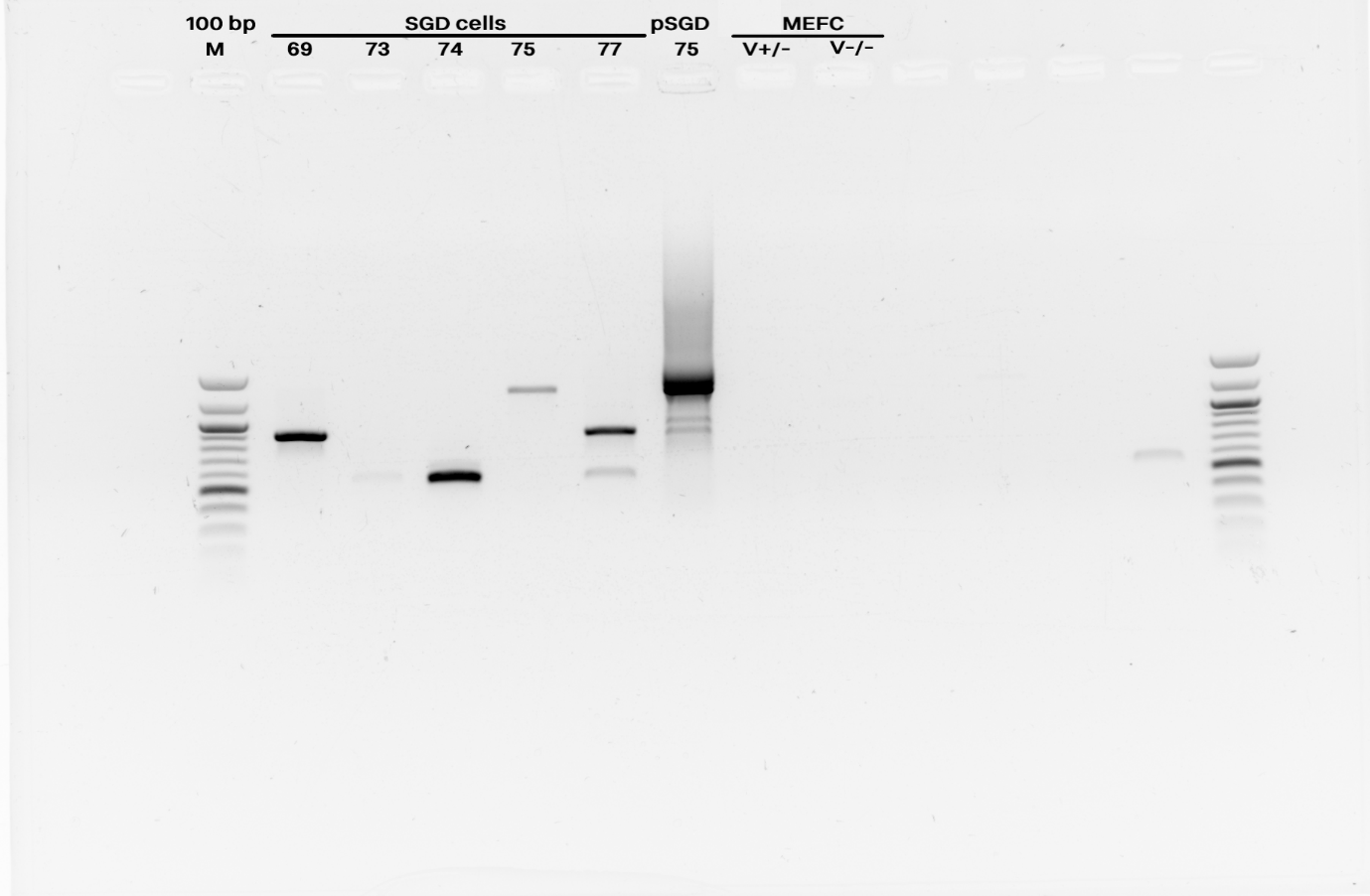


**Figure S12. Full image from a 1.5% agarose gel electrophoresis pertained to the left image in Figure 3C (Puromycin-Venus primers).** The rest part in the right belongs to another experiment.


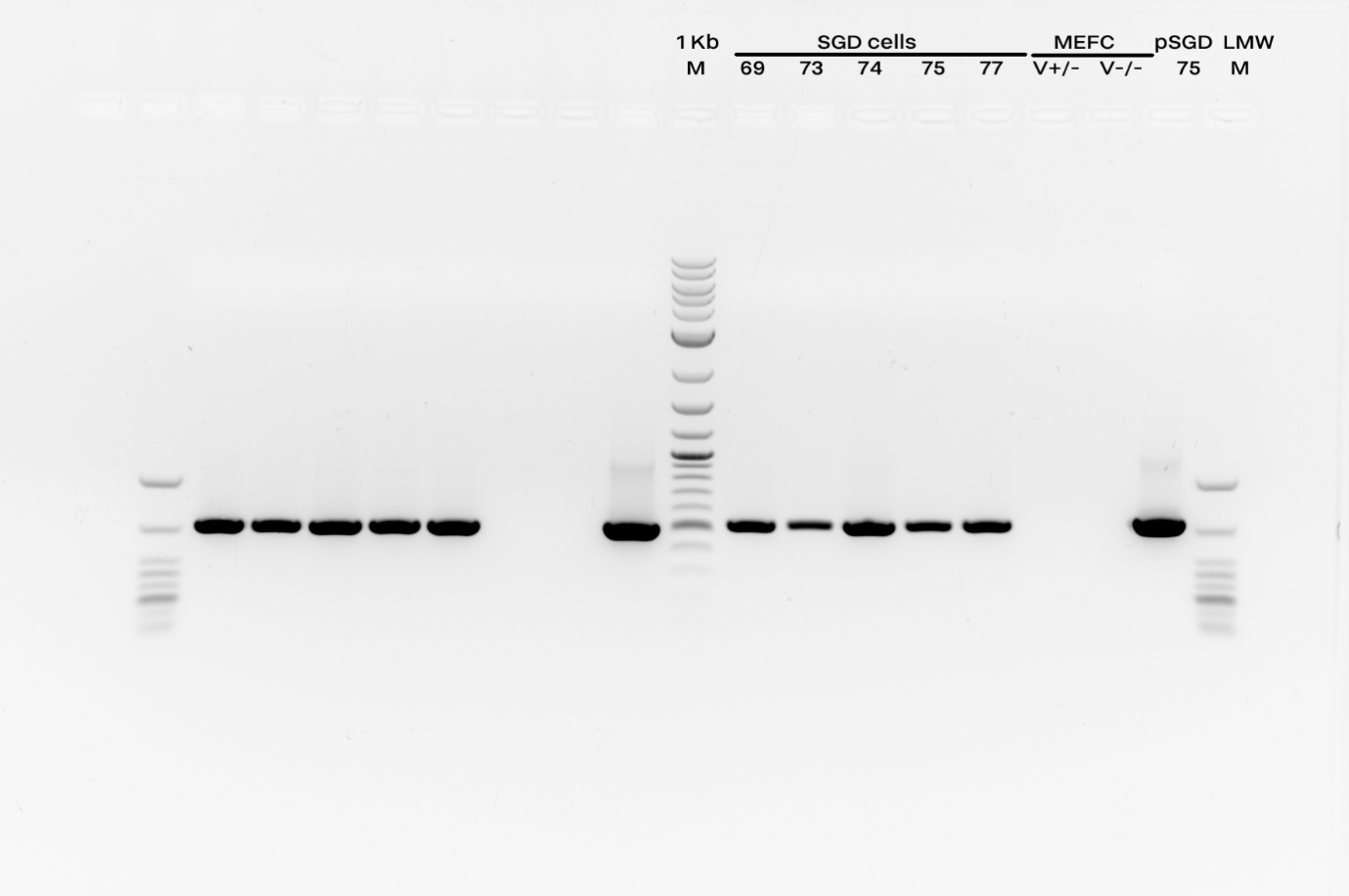


**Figure S13. The full image from a 1% agarose gel electrophoresis belongs to the right cropped image in Figure 3C (Cas9 primers).** The bands in the right part are replicates of the same experiment

**Table S1.** The sequence of gRNAs specific targeting the hybrid intron in the CAGGS promoter (designated with a minus) and the ORF region (designated with a plus) of the Venus transgene. These gRNAs were designed by CRISPOR online software.

| Name | Sequence (5′-3′) |
| --- | --- |
| gRNA-252 | CTTCGCCCGCGCCCGCTAGA |
| gRNA-72 | TTCGGCTTCTGGCGTGTGAC |
| gRNA-69 | GGCTTCTGGCGTGTGACCGG |
| gRNA+121 | GGCGAGGGCGACGCCACCTA |

**Table S2.** Characteristics details of all-in-one plasmids containing the CRISPR/Cas9 system and a large DNA donor.

| No. | Plasmid name | gRNA | Lysozyme | LHA | RHA | LHA to gRNA | RHA to gRNA | Cas9 Promoter | Insertion size | Plasmid size |
| --- | --- | --- | --- | --- | --- | --- | --- | --- | --- | --- |
| 1 | pSGD52 | -252 | No | 1,690 | 1,345 | 294 | 0 | Original | 5,312 | 12,420 |
| 2 | pSGD62 | -252 | Yes | 1,884 | 1,345 | 0 | 0 | Original | 7,115 | 13,435 |
| 3 | pSGD73 | -72 | No | 1,690 | 1,345 | 294 | 0 | Original | 6,475 | 12,420 |
| 4 | pSGD74 | -72 | Yes | 2,064 | 1,345 | 0 | 0 | Original | 7,115 | 13,435 |
| 5 | pSGD75 | -72, +121 | No | 1,977 | 1,208 | 0 | 0 | Hybrid | 6,344 | 12,439 |
| 6 | pSGD77 | -72 | No | 1,977 | 1,208 | 0 | 207 | Hybrid | 5,913 | 12,008 |
| 7 | pSGD69 | -69 | No | 1,977 | 1,208 | 0 | 207 | Hybrid | 5,913 | 12,008 |

**Table S3.** The accessory sequences used for cloning of pSGD plasmids. For the first three accessory sets, each primers set was annealed to make a DNA duplex with overhang strands and were used directly to ligate with the digested backbone vector. The accessory set of U6 promoter-gRNA was used for amplicon of a cassette containing U6 promoter and gRNA in pX459-derived vectors. Both PCR amplicon and backbone vector were digested by Not I and Xba I, and the purified fragments were ligated.

| Accessory set | Name | | Sequence (5′-3′) | Tm | Amplicon length (bp) |
| --- | --- | --- | --- | --- | --- |
| MluI-PacI | | MluI_PacI_FWD | CGCGTTAAGAGCCTACGGCGGTCTAGATTAAT | 61.9 | - |
|  |  | MluI_PacI_REV | TAATCTAGACCGCCGTAGGCTCTTAA | 61.7 |  |
| BbsI-MreI | | BbsI_MreI_FWD | GCTATTGTCTTCCCCCCGGGTTCGAACCG | 61.0 | - |
|  |  | BbsI_MreI_REV | CCGGCGGTTCGAACCCGGGGGGAAGACAA | 62.0 |  |
| AxyI-NotI | | AxyI_NotI_FWD | TCAGGCGTTTAAACATCGATCCTAGGGC | 62.0 | - |
|  | | AxyI_NotI_REV | GGCCGCCCTAGGATCGATGTTTAAACGCC | 62.0 |  |
| U6 promoter-gRNA | | NotI_gRNAx_XbaI_FWD | TTGCTCAGCGGCCGCTACATGTGAGGGCC | 68.6 | 135 |
|  |  | NotI_gRNAx_XbaI_REV | GGTACCTCTAGAGCCATTTGTCTGCAG | 61.3 |  |

**Table S4.** Primers for amplification and detection of Venus transgene

| Primer set | | Name | Sequence (5′-3′) | Tm | Amplicon length (bp) |
| --- | --- | --- | --- | --- | --- |
| LTR SV40 PolyA | LTR SV40_FWD | | CAGGGTTTTCCCAGTCACGA | 59.9 | 508 |
|  | LTR SV40_REV | | TAAATCCTCGAGTGCGTCCAG | 59.9 |  |
| LTR CMV Promoter | LTR CMV_ FWD | | GGCTATGAACTAATGACCCCGT | 59.9 | 526  3199 Venus  7.4-10.3 kb SGD  Venus 0  2 kb SGD  Venus 0  1.8-4.7 kb SGD |
|  | LTR CMV_ REV | | CGCCAAGCTCGAAATTAACCC | 60.2 |  |
| SGD Total | SGD_Total_F | | TGGCAAGTCAGTTAGGACATCT | 60.1 |  |
|  | SGD_Total_R | | TCCCTGTCTTAGGTCAGTTAGGA | 62.9 |  |
| SGD_Puromycin | SGD_Total_F | | TGGCAAGTCAGTTAGGACATCT | 60.1 |  |
|  | SGD_Puro_R | | CGCCACACCGTCGATCC | 59.8 |  |
| SGD_Cas9 | SGD_Cas9_F | | AGGCCATTCTTCTTCTCGCC | 60.5 |  |
|  | SGD_Total_R | | TCCCTGTCTTAGGTCAGTTAGGA | 62.9 |  |
| Late Venus | Late Venus- FWD | | ACGCGTTAAGATACATTGATGAGTT | 59.4 | 556 |
|  | Late Venus- REV | | AGGACGACGGCAACTACAAG | 60.0 |  |
| Early Venus | Early Venus- FWD | | CTTGTAGTTGCCGTCGTCCT | 60.0 | 668 |
|  | Early Venus- REV | | TGCCTTTTATGGTAATCGTGCG | 59.6 |  |
| Total Venus | Late Venus- FWD | | ACGCGTTAAGATACATTGATGAGTT | 59.4 | 1204 |
|  | Early Venus- REV | | TGCCTTTTATGGTAATCGTGCG | 60.0 |  |
| Venus-Lys segment | Venus-Lys- FWD | | GTAACCACTCTCCCATTTGGC | 58.9 | 504 |
|  | Venus-Lys- REV | | ATGGTAATCGTGCGAGAGGG | 59.6 |  |
| Lys-Cas9 segment | Lys-Cas9- FWD | | AAACTGCAAACTACCCAAGAAAT | 57.1 | 520 |
|  | Lys-Cas9- REV | | CGCTGATGCTGTAGCTTGTG | 59.6 |  |
| Puromycin-Venus | Puromycin-Venus- FWD | | CTTGTAGTTGCCGTCGTCCT | 60.1 | 549 |
|  | Puromycin-Venus- REV | | CAGCCATCTGTTGTTTGCCC | 60.0 |  |
| Cas9 segment | Cas9- FWD | | GGTAGCCTTGCCGATTTCCT | 60.1 | 407 |
|  | Cas9- REV | | ACAATCTGACCAAGGCCGAG | 60.0 |  |
|  |  | |  |  |  |
|  |  | |  |  |  |

**Table S5.** Off-target sequences of CAGGS-targeting gRNAs on the mouse genome. The results was provided by the online webtool of Cas-OFFinder (<http://www.rgenome.net/cas-offinder/>), considering up to 3 mismatches. Mismatches between gRNAs and the mouse genome are depicted with lowercase letters.

| gRNA name | No. of off-targets | Off-target sequence | Chromosome | Position | Mismatches |
| --- | --- | --- | --- | --- | --- |
| gRNA-72 | 2 | gRNA: TTCGGCTTCTGGCGTGTGAC NGG   DNA: TTCGGtTTCTGGgGTGgGAC AGG | Chr7 | 19159866 | 3 |
|  |  | gRNA: TTCGGCTTCTGGCGTGTGAC NGG   DNA: TTCGGCTTtTaGCGTGTGtC TGG | Chr3 | 78933578 | 3 |
| gRNA-252 | 1 | gRNA: CTTCGCCCGCGCCCGCTAGA NGG   DNA: CTTCGCCCtCGCCCcCgAGA CGG | Chr5 | 99977950 | 3 |
| gRNA-69 | 1 | gRNA: GGCTTCTGGCGTGTGACCGG NGG   DNA: GGCTTCTGGCtTGTcACCaG AGG | Chr1 | 165706449 | 3 |

**Table S6.** Primer-probe assays used for the detection of Venus target and Cas9 inserted gene.

| Primer set | Name | | Sequence (5′-3′) | Tm | Amplicon length (bp) |
| --- | --- | --- | --- | --- | --- |
| Primer-Probe-Assay-1 | | Venus-FWD | CCCTTCAGCTCGATTCTGTT | 58.4 | 102 |
|  |  | Venus-REV | GCAGGAGAGAACCATCTTCTT | 59.5 |  |
|  |  | Venus-Probe | CCTCGGCTCTGGTCTTGTAGTTGC | 68.5 |  |
| Primer-Probe-Assay-2 | | Cas9-FWD | GTTCCTGGTCCACGTACATATC | 62.1 | 135 |
|  |  | Cas9-REV | GGATCGAAGAGGGCATCAAA | 58.4 |  |
|  |  | Cas9-Probe | TTCTTTCAGGATCTGGCTGCCCAG | 66.9 |  |

**Supplementary methods**

The cloning details for construction of large plasmids (SGD) inculding the insertion cassette of CRISPR/Cas9 system and puromycin with or without lysozyme as well as the left homology arm (CAGGS promter) and right homology arm (Venus CDS and SV40 polyA).

**Making pSGD62 and pSGD74 constructs:** First, we made the pSGD62 and pSGD72 plasmids (13.4 kb) which contain the pT2-Venus backbone, human lysozyme CDS, as well as gRNA, Cas9, and puromycin-encoding cassette which was cut from either pX459-252 or pX459-72 plasmid. The cloning steps were as followed: cutting pT2_Venus with Fastdiget MreI (ThermoFisher) and then HF-PacI (NEB) restriction enzymes, extraction of the 5.7 kb backbone using the MN kit from a 0.7 % agarose gel, digestion of the synthetic stock using PciI (10x Buffer Tango) and then MreI (FastDigest, ThermoFisher), extraction of the 1.1 kb fragment from the gel, digestion of pX459-252 /pX459-72 using PciI (10x Buffer Tango) and then HF-NotI (NEB), extraction of the 6.4 kb fragment from the gel, annealing of two accessory oligos (5 nM) containing NotI and PacI overhangs (Table 3), and making the SGD construct via simultaneous ligation of four fragments in a single tube. All restriction enzymes were inactivated by heating at 80°C for 15 min. The ligation reaction included Venus backbone (60 ng), lysozyme fragment (40 ng), CRISPR/Cas9 fragment (100 ng), accessory oligos (1 µl), 10X T4 DNA ligase buffer, T4 DNA ligase (2 µl) in 30 µl final volume. Ligation was carried out through a 1 h incubation at room temperature. The cloned plasmids were transformed into DH5-Alpha cells followed by culture on the agar plates containing ampicillin. Single colonies were picked up and using for a liquid culture. Plasmids were extracted with a miniprep kit (Genejet Plasmid miniPrep kit, #K0502) and digested with Mre I and Pac I. Plasmids containing the desired bands were sent for sequencing (LGC Genomics, Germany).

**Making pSGD52 and pSGD73 constructs:** Plasmids pSGD62 and pSGD74 were digested with Mre I and PciI restriction enzymes and the backbone plasmid was extracted from the gel. Two accessory oligonucleotides which were complementary to each other with specific overhangs complementary to the staggered sequences in the backbone plasmids were synthesized (Table S3). Accessory oligos were diluted in the annealing buffer (10mM Tris-HCl pH 8.0, 1mM EDTA, and 100Mm NaC) ^1^, and ligated into the backbone vectors. These SGD plasmids (12.4 kb) did not have a 370 bp-segment of the left homology arm (LHA) and the lysozyme cDNA.

**Making pSGD75 and pSG76 constructs:** Plasmid pSGD74 which includes lysozyme transgene under CAGGS promoter followed by the gRNA-72 was digested with NotI-HF (NEB, Germany) and AxyI-HF (NEB, Germany) overnight. The 13.1 kb fragment (50 ng) was ligated with the digested synthetic fragment (950 bp) included gRNA-72 and gRNA+121 (150 ng) for 2 h at room temperature. The new construct, namely pSGD76 (14.5 kb), was digested with MreI (FastDigest, ThermoFisher) and Age I-HF (NEB) in a mixture of 0.5 X from their specific 10X buffer for overnight. The digested fragment (11.5 kb) was ligated with the digested synthetic hybrid promoter (950 bp) for 2 h at room temperature. The new construct was named pSGD75 (12.4 kb).

**Making pSGD77 construct:** Plasmid pSGD75 which included gRNA-72 and gRNA+121 was digested with SfuI restriction enzyme (#11243497001, Roche, Germany) at 37°C for overnight. The linearized fragment was extracted from a 0.5 % agarose gel, and re-ligated to make the pSGD77 plasmid (12.0 kb) containing only gRNA-72.

**Making pSGD69 construct:** Plasmid pSGD77 which included gRNA-72 was digested with NotI-HF and XbaI-HF restriction enzymes (Roche, Germany) at 37°C for overnight to remove the sequence of U6 promoter and gRNA-72. The linearized fragment was extracted from a 0.5 % agarose gel. Using the NotI_gRNAx_XbaI_FWD and REV primers (Table S3), the sequence containing U6 promoter and gRNA-69 was amplified from the plasmid source, digested with NotI-HF and XbaI-HF restriction enzymes, purified by gel extraction kit, and ligated into the linearized pSGD77 fragment using the T4 DNA Ligase to make the pSGD69 plasmid (12.0 kb).

1. Guo Y, Perez AA, Hazelett DJ, et al. CRISPR-mediated deletion of prostate cancer risk-associated CTCF loop anchors identifies repressive chromatin loops. Genome biology 2018;19(1):160.
